# GM-CSF Inhibits c-Kit and SCF Expression by Bone Marrow-Derived Dendritic Cells

**DOI:** 10.3389/fimmu.2017.00147

**Published:** 2017-02-16

**Authors:** Amairelys Belen Barroeta Seijas, Sonia Simonetti, Sara Vitale, Daniele Runci, Angela Caterina Quinci, Alessandra Soriani, Mattia Criscuoli, Irene Filippi, Antonella Naldini, Federico Maria Sacchetti, Umberto Tarantino, Francesco Oliva, Eleonora Piccirilli, Angela Santoni, Francesca Di Rosa

**Affiliations:** ^1^Institute of Molecular Biology and Pathology, National Research Council (CNR), c/o Department of Molecular Medicine, University of Rome “Sapienza”, Rome, Italy; ^2^Department of Molecular Medicine, University of Rome “Sapienza”, Rome, Italy; ^3^Istituto Pasteur Italia – Fondazione Cenci Bolognetti, Rome, Italy; ^4^Department of Molecular and Developmental Medicine, University of Siena, Siena, Italy; ^5^Centro Traumatologico Ortopedico Andrea Alesini Hospital, Rome, Italy; ^6^Department of Orthopaedics and Traumatology, University of Rome “Tor Vergata”, Rome, Italy

**Keywords:** bone marrow-derived dendritic cells, conventional dendritic cell subsets, bone marrow, dendritic cell survival, dendritic cell homeostasis, GM-CSF, stem cell factor

## Abstract

Stem cell factor (SCF), the ligand of c-kit, is a key cytokine for hematopoiesis. Hematopoietic precursors express c-kit, whereas differentiated cells of hematopoietic lineage are negative for this receptor, with the exception of NK cells, mast cells, and a few others. While it has long been recognized that dendritic cells (DCs) can express c-kit, several questions remain concerning the SCF/c-kit axis in DCs. This is particularly relevant for DCs found in those organs wherein SCF is highly expressed, including the bone marrow (BM). We characterized c-kit expression by conventional DCs (cDCs) from BM and demonstrated a higher proportion of c-kit^+^ cells among type 1 cDC subsets (cDC1s) than type 2 cDC subsets (cDC2s) in both humans and mice, whereas similar levels of c-kit expression were observed in cDC1s and cDC2s from mouse spleen. To further study c-kit regulation, DCs were generated with granulocyte-macrophage colony-stimulating factor (GM-CSF) from mouse BM, a widely used protocol. CD11c^+^ cells were purified from pooled non-adherent and slightly adherent cells collected after 7 days of culture, thus obtaining highly purified BM-derived DCs (BMdDCs). BMdDCs contained a small fraction of c-kit^+^ cells, and by replating them for 2 days with GM-CSF, we obtained a homogeneous population of c-kit^+^ CD40^hi^ MHCII^hi^ cells. Not only did BMdDCs express c-kit but they also produced SCF, and both were striking upregulated if GM-CSF was omitted after replating. Furthermore, a small but significant reduction in BMdDC survival was observed upon SCF silencing. Incubation of BMdDCs with SCF did not modulate antigen presentation ability of these cells, nor it did regulate their membrane expression of the chemokine receptor CXCR4. We conclude that the SCF/c-kit-mediated prosurvival circuit may have been overlooked because of the prominent use of GM-CSF in DC cultures *in vitro*, including those human DC cultures destined for the clinics. We speculate that DCs more prominently rely on SCF *in vivo* in some microenvironments, with potential implications for graft-versus-host disease and antitumor immunity.

## Introduction

Dendritic cells (DCs) are cells of hematopoietic origin that develop in the bone marrow (BM) and play a key role in immunity and tolerance. Resting DCs are scattered as sentinels in tissues throughout the body, contributing to maintain tolerance in healthy conditions (1, 2). Upon pathogen entry, tissue DCs become activated, uptake and process antigens, and acquire an increased capacity to migrate to draining lymph nodes (LNs). Changes in migratory behavior of activated DCs are reflected by modulation of chemokine receptors, for instance, upregulation of CCR7, the receptor for CCL19/21, and of CXCR4, the receptor for CXCL12 (3–6). Fully activated or mature DCs act as professional antigen-presenting cells (APCs), with the unique ability to prime naïve CD4 and CD8 T cells (2, 7). Furthermore, DCs orchestrate immune response by producing a wide array of membrane molecules, cytokines, and chemokines (8). After activation, DCs are destined to die by apoptosis or necroptosis, depending on the maturating stimuli (9, 10). DC survival is also regulated by several inflammatory cytokines, including type I interferon (IFN) and IL-15 (11, 12).

Distinct DC subsets have been identified (2, 13). Classical or conventional DCs (cDCs) are specialized in antigen processing and presentation, whereas plasmacytoid DCs (pDCs) produce high levels of type I IFN in response to viral infection (2). In mice, cDCs express high levels of CD11c integrin, while pDCs have an intermediate expression of this marker (14). At least two cDC subsets have been identified: the most abundant type 2 cDC subset (cDC2) expresses CD11b in mice and CD1c in humans; the type 1 cDC subset (cDC1) expresses CD8α and/or CD103 in mice and CD141 in humans (14). Under pathological conditions, novel DC subsets could appear, for example, monocyte-derived inflammatory DC (15). Different sets of chemokine receptors are expressed by DC subsets, for example, CX3CR1, the receptor for fractalkine, is expressed by pDCs, human blood cDC2, and kidney DCs (16–18).

Peripheral DCs have a finite life span (19). DCs mostly derive from common myeloid progenitors (CMPs) in the BM, with some contribution by common lymphoid precursors (2). Both FMS-like tyrosine kinase 3 ligand (Flt3-L) and granulocyte-macrophage colony-stimulating factor (GM-CSF) regulate DC development and maintenance of normal differentiated DC numbers (20–22). In the absence of GM-CSF, cDC numbers in spleen and LN were decreased about 2- and 4-fold, respectively; cDC reduction was more prominent in Flt3-L ko mice, and pDCs were also greatly impaired in these mice (21). Data in Flt3 ko and Flt3/GM-CSF receptor double ko mice supported a specific role for Flt3 signaling in cDC maintenance (23). Notably, some residual cDCs were found in the spleen and BM of Flt3-L ko mice, and they were still present in Flt3-L/GM-CSF double ko mice (21). Altogether, these results suggest that some unknown mechanisms might contribute to differentiated DC survival in lymphoid organs (21).

Dendritic cells can express c-kit, the receptor for stem cell factor (SCF) (13, 24). In a mouse model of allergic asthma, it was found that c-kit was expressed by DCs from lung of mice immunized intranasally with OVA/cholera toxin or OVA/CpG, but not by lung DCs from untreated mice (25). Activation of SCF/c-kit axis in DC promoted Th2 and Th17 but not Th1 response (25–27). Under physiological conditions, lymphoid-tissue cDCs but not pDCs express c-kit (13, 14, 28). Despite increasing knowledge of location-specific DC characteristics, several questions regarding c-kit expression by DCs remain, for instance whether DCs from different lymphoid organs display differences in c-kit expression, and whether SCF binding to c-kit plays a role in DC homeostasis particularly in those organs wherein SCF is highly expressed, such as the BM (29).

In the BM, fibroblasts, endothelial cells, and other cell types produce SCF, an essential cytokine for hematopoiesis, that is also required for germ cell and melanocyte development (29–32). Soluble and membrane isoforms of SCF are generated as a result of alternative splicing and proteolytic cleavage (33). Binding of SCF to its membrane receptor c-kit (also called CD117) activates intracellular signaling molecules including PI-3 kinase, PLCγ, src-family kinases, and regulating cell survival, proliferation, adhesion (32–34).

In this paper, we evaluated c-kit expression by DC subsets in mouse and human BM and studied SCF/c-kit axis in DCs generated *in vitro* from mouse BM.

## Materials and Methods

### Cytokines and Culture Media

Recombinant mouse SCF and Flt3-L were purchased from Immunotools (Friesoythe, Germany), recombinant mouse GM-CSF from Peprotech (Rocky Hill, NJ, USA). Opti-MEM Medium (Thermo Fisher Scientific, Waltham, MA, USA) was supplemented with glutamine, penicillin/streptomycin, 50 µM β-mercaptoethanol (Complete Opti-MEM medium). Complete Opti-MEM medium was not supplemented with any serum, except in the cultures with OT-1 and OT-2 cells, as indicated. RPMI Medium 1640 (Sigma-Aldrich, Milan, Italy) was supplemented as above, plus 10% heat-inactivated fetal calf serum (FCS) (complete RPMI medium). Opti-MEM is an optimized version of MEM containing insulin and transferrin, but does not contain GM-CSF, Flt3-L, SCF, or other cytokines (personal communication from Thermo Fisher Scientific Technical Support).

### Mouse Sample Collection and Preparation

Female C57BL/6J (B6) and OT-2 TCR transgenic mice were purchased from Charles River and housed at the animal facility of Istituto Superiore di Sanità of Rome (ISS), according to institutional guidelines (DL116/92 and 26/2014). Female OT-1 TCR transgenic mice were kindly provided by Dr. M. R. Castrucci (ISS). The OT-1 transgenic TCR recognizes the Kb-restricted OVA 257–264 peptide (35), while the OT-2 transgenic TCR recognizes the I-Ab-restricted OVA 323-339 peptide (36). CX3cr1^gfp/+^ and CX3cr1^gfp/gfp^ B6 mice were purchased from JAX Mice and Services (Bar Harbor, ME, USA) (37). Mice were sacrificed at 5–16 weeks of age and spleen, peripheral, and mesenteric LNs and BM obtained as we previously described (38, 39). In some experiments, CD11c^+^ cells were enriched from either spleen or BM with anti-CD11c magnetic microbeads (Miltenyi Biotec, Bergisch Gladbach, Germany).

### BM-Derived DCs (BMdDCs)

We generated DCs from BM cells as previously described (40, 41), with few modifications. Briefly, 10–15 × 10^6^ BM cells were cultured in complete RPMI medium with 20 ng/ml of GM-CSF in Petri dishes (BD Falcon, BD Biosciences, San Jose, CA, USA). After 3 days, fresh medium with GM-CSF was added. At day 7, we collected non-adherent and slightly adherent cells after detachment with PBS 3 mM EDTA. CD11c^+^ cells were purified with anti-CD11c magnetic microbeads (Miltenyi Biotec), thus obtaining BMdDCs. In some experiments, DCs were generated by culturing BM cells with Flt3-L at 100 ng/ml for 8 days, as previously described (42).

### Antigen Presentation Assay

OT-1 and OT-2 mice were sacrificed and single cell suspensions were obtained from spleen and LNs. OT-1 CD8^+^ and OT-2 CD4^+^ cells (≥87% pure) were obtained after incubation with anti-CD8β.2 fluorescein isothiocyanate (FITC) monoclonal antibody (mAb) (BD) and CD4 FITC mAb (BD), respectively, followed by positive selection with anti-FITC magnetic microbeads (Miltenyi Biotec). Cells were labeled for 8 min at room temperature with 2.5 µM carboxyfluorescein diacetate succinimidyl ester (CFSE, Molecular Probes, Eugene, OR, USA). For antigen presentation assays, BMdDCs were kept for 2 days in complete Opti-MEM medium with GM-CSF at 20 ng/ml, then incubated for 5 h with Ovalbumin (OVA, Hyglos GmbH, Resenburg, Germany) at 0.2 mg/ml, in the presence or not of SCF at 100 ng/ml. After extensive washings, BMdDCs (200–250 × 10^3^ cells/well) were cocultured in flat-bottom 96-well plates in complete Opti-MEM medium with 5% FCS with either purified CFSE-labeled OT-1 CD8^+^ (700–750 × 10^3^ cells/well) or purified CFSE-labeled OT-2 CD4^+^ (200 × 10^3^ cells/well) cells for 3 days. CFSE dilution by TCR^+^ CD8^+^ and TCR^+^ CD4^+^ cells was evaluated by flow cytometry (43–45).

### Membrane Staining and Flow Cytometry

Cell membrane staining was performed with fluorochrome-conjugated mAbs, after blocking with anti-FcγR (clone 2.4G2) mAb. The following mAbs were used (clone indicated in parentheses): anti-CD11c (HL3), anti-I-Ab or MHCII (M5/114.15.2) anti-CD40 (HM40-3), anti-CD11b (M1/70), anti-CD8α (53-6.7), anti-CD3 (145-2C11), anti-NK1.1 (PK136), anti-c-kit (2B8), anti-TCRβ (H57-597); anti-CD4 (RM4-4); anti-CD8β.2 (53-5.8); anti-CXCR4 (2B11) [from BD Biosciences; Biolegend, San Diego, CA, USA; Miltenyi Biotec; eBioscience; conjugated with FITC, phycoerythrin (PE), peridinin chlorophyll protein (PerCP)-Cy5.5, PE-Cy7, Alexa 647, APC, APC-Vio770; APC-H7]. Dead cells were excluded with Propidium Iodide (PI, Sigma-Aldrich). Samples were analyzed by FACSCanto I and II (BD Biosciences). Data were analyzed using FlowJo software, v.9.7.6 (FlowJo, Ashland, OR, USA).

### Real-time PCR

Total RNA was extracted by TriReagent (Sigma-Aldrich). One microgram of total RNA was used for cDNA first-strand synthesis according to the manufacturer’s protocol for Moloney MLV reverse transcriptase (Promega, Madison, WI, USA). Real-time PCR was performed using the ABI Prism 7900 sequence detection system (Applied Biosystems, Foster City, CA, USA). cDNAs were amplified in triplicate with primers for c-kit (Mm00445212_m1), SCF (Mm00442972_m1), and hprt-1 (Mm00446968 m1) (Applied Biosystems, Thermo Fisher Scientific), all conjugated with fluorochrome FAM. Relative expression of each gene versus hprt-1 was calculated according to the 2^−∆∆Ct^ method.

### Western Blot

Immunoblotting was performed as we previously described, after cell lysis with 1% Triton X-l00, 0.1% SDS Tris buffer containing protease inhibitor cocktail (Sigma-Aldrich) and the phosphatase inhibitors NaF, Na_3_VO_4_, phenylmethylsulfonyl fluoride (46). Equal amounts of proteins were separated by 10% SDS-PAGE, transferred onto PVDF membrane by Trans-Blot TurboTM Transfer System (Bio-Rad Laboratories), and probed with anti-phospho-AKT (Ser 473), anti-AKT and anti-β-actin antibodies (Cell Signaling Technology, Danvers, MA, USA). Chemiluminescence detection was performed by a CCD camera gel documentation system (ChemiDocXRS, Bio-Rad Laboratories, Hercules, CA, USA).

### ELISA

BM-derived DCs were lysed as above without the phosphatase inhibitors. BMdDC culture supernatants (100 µl/well) and BMdDC lysates (25 µg of cell lysate/well) were tested by mouse SCF ELISA kit (Boster Immunoleader, Pleasanton, CA, USA).

### SCF Silencing

Stem cell factor silencing was performed by transfecting BMdDCs with Lipofectamine^®^ RNAiMax reagent (Invitrogen, Thermo Fisher Scientific) using the following siRNAs: SCF-siRNA (sc-39735, consisting of a pool of three target-specific 19–25 nt siRNA), scrambled control-A siRNA (sc-37007), fluorescein conjugated control-A siRNA (sc-36869), all from Santa Cruz (Dallas, TX, USA).

### Cell Viability Assay by Flow Cytometry

Stem cell factor- or control-silenced BMdDCs were cultured for 2 days in 96-well plates at 2 × 10^5^/well in complete Opti-MEM medium with or w/o GM-CSF at 20 ng/ml. Cells were then collected, stained with Annexin V FITC and PI, and analyzed by flow cytometry. In experiments with blocking anti-c-kit mAb, BMdDCs were cultured as above in the presence of 10 µg/ml of either anti-c-kit (ACK2) or isotype control (RTK4530) mAb (both Low Endotoxin Azide Free, Biolegend). After 2 days, cells were collected and analyzed by Annexin V FITC/PI as above.

### Cell Number Determination

BM-derived DC numbers were determined by the CyQUANT Cell Proliferation Assay kit from Invitrogen according to manufacturer’s instructions. Optical density was determined using a VICTOR X Multilabel Plate Reader.

### Human BM Samples Collection and Flow Cytometric Analysis

Pieces of spongy bone, which would otherwise have been discarded, were obtained from systemically healthy patients undergoing hip replacement at either Centro Traumatologico Ortopedico Andrea Alesini Hospital or Policlinico Tor Vergata, Rome, after approval by Institutional Ethics Committee, and informed consent from patients in accordance with the Declaration of Helsinki (study no. 129.14, prot 76699 and study no. 156.15, prot. 0030053/2015). BM mononuclear cells were isolated by Lympholyte-H (Cedarlane, NC, USA) density-gradient centrifugation. Flow cytometry was performed as above, using FcR Blocking Reagent and Dead Cell Discrimination Kit (Mitenyi Biotec) before addition of the following fluorochrome-conjugated mAbs: CD1c or BDCA-1 (AD5-8E7), CD141 or BDCA-3 (AD5-14H12), CD45 (2D1) CD14 (TÜK4), CD19 (LT19), c-kit (104D2) [from BD Biosciences; Miltenyi Biotec; Exbio, Prague, Czech Republic; conjugated with phycoerythrin (PE), PerCP-Vio700, PE-Cy7, APC, APC-H7]. Cells were fixed with PBS formaldehyde 1.2% before flow cytometric analysis.

### Statistics

We performed a two-tailed paired Student’s *t*-test using Prism v.6.0f, GraphPad Software (La Jolla, CA, USA). Differences were considered significant when *P ≤ 0.05, **P ≤ 0.01, ***P ≤ 0.001.

## Results

### c-Kit Is Expressed by a Fraction of DCs in the BM of WT Mice

**Figure 1:**
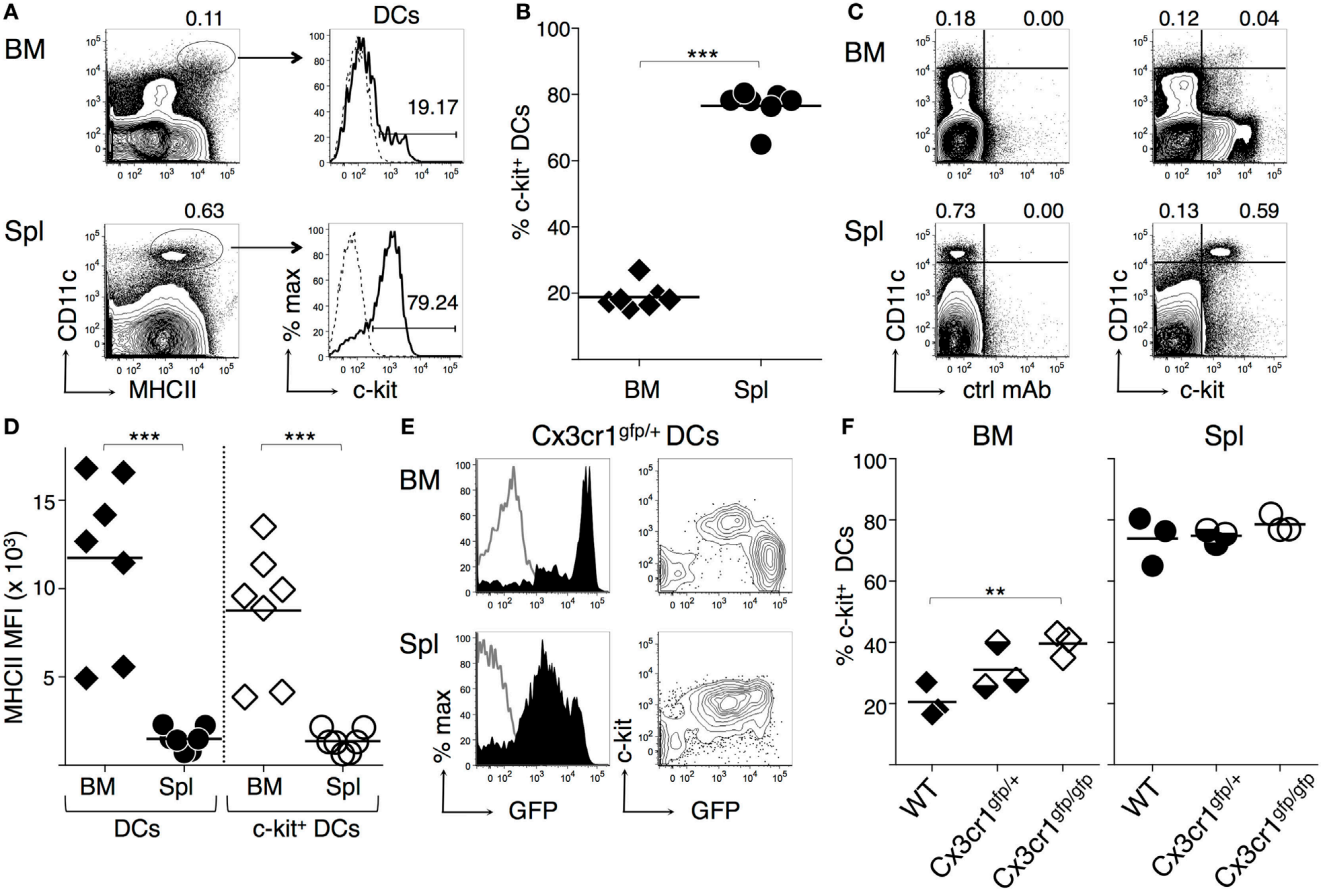
**c-Kit membrane expression by bone marrow (BM) and spleen dendritic cells (DCs)**. BM and spleen cells were obtained from untreated mice, stained with fluorochrome-conjugated monoclonal antibodies (mAbs), and analyzed by flow cytometry (for gating strategy see Figure S1 in Supplementary Material). **(A–D)** DCs from wild-type C57BL6/J (B6) mice. **(A)** Typical flow cytometric profiles, showing c-kit^+^ cell percentages among CD11c^high^ MHCII^+^ DCs. In the histograms, solid lines represent c-kit staining profiles, dashed lines isotype control mAb. Numbers represent percentages of cells in the indicated regions. **(B)** Summary of results obtained from BM and spleen of individual mice and average values of each organ (bar). Percentages of c-kit^+^ cells were obtained after subtracting background values with isotype control mAb. **(C)** Representative dot plots showing c-kit expression by CD11c^high^ cells. The numbers represent percentages of cells in the indicated upper left and upper right regions. **(D)** MHC-II median fluorescence intensity (MFI) in DCs and c-kit^+^ DCs from either BM or spleen, gated as in **(A)**. Summary of results obtained from BM and spleen of individual mice and average values of each organ (bar). **(E–F)** DCs from Cx3cr1-genetically modified mice. **(E)** Typical flow cytometric profiles, showing c-kit and green fluorescence protein (GFP) expression by CD11c^high^ MHCII^+^ DCs from Cx3cr1^gfp/+^ mice, gated as in **(A)**. In the histograms, filled lines represent Cx3cr1^gfp/+^ DCs, empty lines WT DCs. **(F)** Summary of results obtained from BM and spleen of individual Cx3cr1^gfp/+^, Cx3cr1^gfp/gfp^, and WT mice and average values of each group (bar). Percentages of c-kit^+^ cells were obtained after subtracting background values with isotype control mAb. See Figure S2 in Supplementary Material for typical DC flow cytometric profiles of Cx3cr1-genetically modified mice. In **(A,C,E)** representative data of *N* = 3–7 in 3–6 experiments, in **(B,D)** *N* = 7 in six experiments, in **(F)** *N* = 9 in three experiments (1 mouse/group in each experiment) (***P ≤ 0.001; **P ≤ 0.01).

We analyzed membrane c-kit expression by BM and spleen DCs freshly obtained from untreated C57BL6/J (B6) mice, gating on CD11c^hi^ MHC-II^+^ DC as previously described (47). Typical flow cytometry profiles are shown in Figure 1A (see Figure S1A in Supplementary Material for our gating strategy, based on forward and side scatter plots and exclusion of CD3^+^, NK1.1^+^, and dead cells). While the great majority of spleen DCs expressed membrane c-kit, only a small percentage of BM DCs was c-kit^+^ (Figures 1A,B). Results were similar after about 100-fold enrichment of CD11c^+^ cells by immunomagnetic selection (Figures S2A,B in Supplementary Material). c-kit^+^ DCs were characterized by intermediate levels of membrane c-kit, that is <10^4^ fluorescence intensity, whereas other cells in spleen and BM were c-kit^hi^ (Figure 1C). We observed that MHC-II median fluorescence intensity (MFI) was higher in BM DCs than in spleen DCs, in agreement with previous results (47). Furthermore, MHC-II MFI was higher in c-kit^+^ BM DCs than in c-kit^+^ spleen DCs (Figure 1D).

### c-Kit and High CX3CR1 Expression by BM DCs Are Inversely Correlated in a CX3CR1-GFP Reporter Mouse

It was previously shown that most BM DCs are characterized by high CX3CR1 expression, using a mouse model in which green fluorescence protein (GFP) replaces CX3CR1 by gene targeting (47). We analyzed BM DCs from CX3cr1^gfp/+^ mice by flow cytometry to investigate c-kit expression in respect to GFP intensity. We found that c-kit was expressed by the few intermediate green but not by the large population of bright green BM DCs from CX3cr1^gfp/+^ mice (Figure 1E), whereas in the spleen most CX3cr1^gfp/+^ spleen DCs were c-kit^+^ and had intermediate green fluorescence intensity (Figure 1E). Furthermore, CX3CR1-deficient CX3cr1^gfp/gfp^ BM DCs contained a significantly higher percentage of c-kit^+^ cells than WT BM DCs, while the percentage of c-kit^+^ cells within spleen DCs was similar in WT, CX3cr1^gfp/+^, and CX3cr1^gfp/gfp^ mice (Figure 1F; Figures S2C,D in Supplementary Material). These results suggest that c-kit expression and high levels of CX3CR1 are inversely correlated in BM DCs and that the presence of c-kit^+^ DCs in the BM does not depend on CX3CR1 expression.

### c-Kit Is Expressed by All cDC1s and by a Fraction of cDC2s in the BM of WT Mice

**Figure 2:**
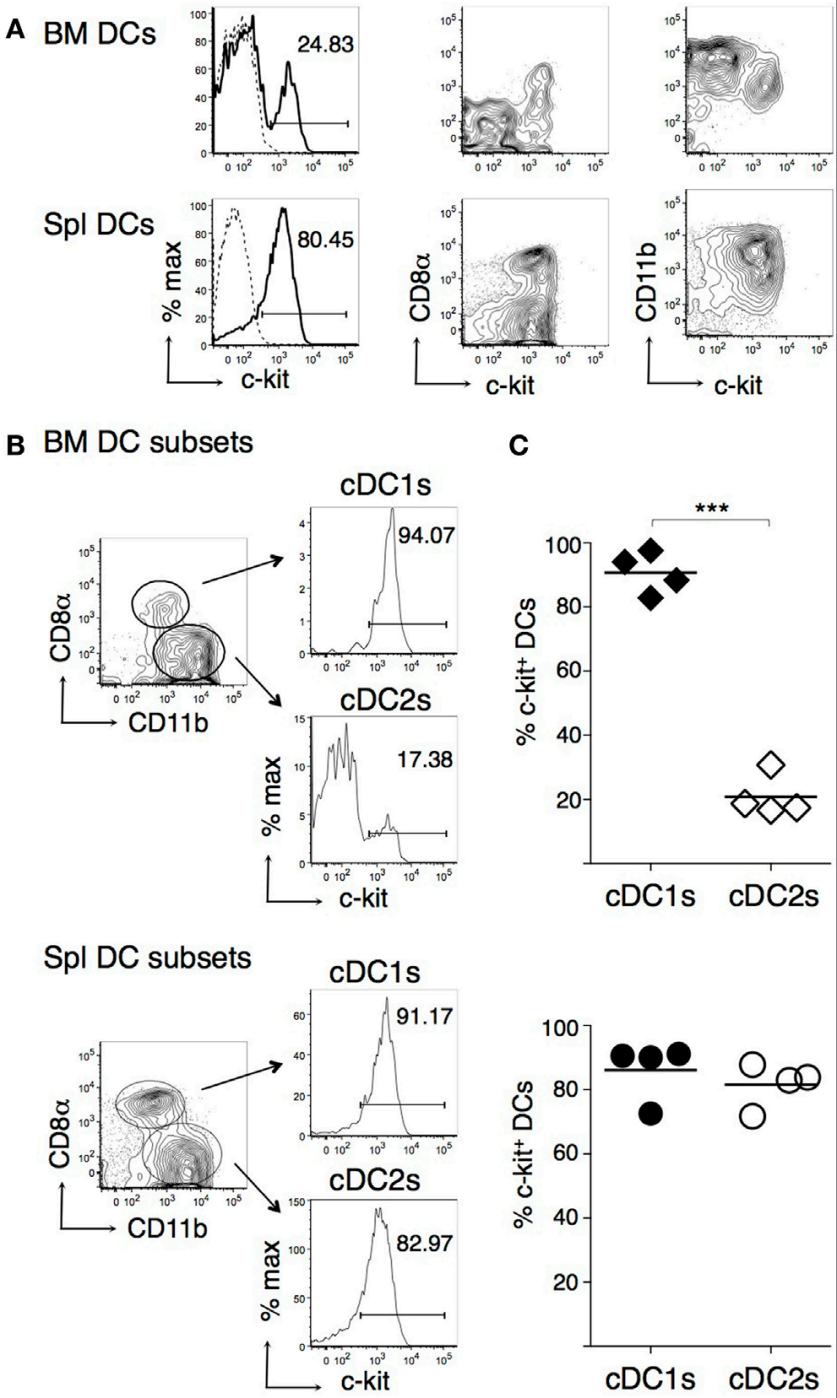
**c-Kit membrane expression by bone marrow (BM) and spleen type 1 cDC subsets (cDC1s) and type 2 cDC subsets (cDC2s)**. BM and spleen cells were obtained from untreated B6 mice, stained with fluorochrome-conjugated monoclonal antibodies (mAbs), and analyzed by flow cytometry. CD11c^high^ MHCII^+^ dendritic cells (DCs) were gated for analysis as in Figure 1A. **(A)** Typical flow cytometric profiles, showing c-kit, CD8α, and CD11b expression by DCs. In histograms, solid lines represent c-kit staining profiles, dashed lines represent isotype control mAb. The numbers represent percentages of cells in the indicated regions. **(B)** Representative histograms showing percentages of c-kit^+^ cells among cDC1s and cDC2s, gated as shown. **(C)** Percentages of c-kit^+^ cells among cDC1s and cDC2s from either BM or spleen of individual mice and average values of each organ (bar). In **(A,B)** representative data of *N* = 4 in three experiments, panel **(C)** *N* = 4 in three experiments (***P ≤ 0.01).

We then performed flow cytometric analysis of cDC subsets from spleen and BM of WT mice, by gating on either cDC1s or cDC2s, based on CD8α and CD11b expression (14, 48). We found that virtually all cDC1s and a small proportion of cDC2s expressed c-kit in the BM, whereas both cDC subsets were c-kit^+^ in the spleen (Figures 2A,B). The difference in c-kit percentages between BM cDC1s and cDC2s was statistically significant (Figure 2C). In the BM, c-kit^+^ DCs expressed intermediate levels of CD11b, while c-kit^−^ DCs were CD11b^hi^ (Figure 2A, right top panel).

### An *In Vitro* Culture System to Analyze c-Kit Expression by Mouse BMdDCs

**Figure 3:**
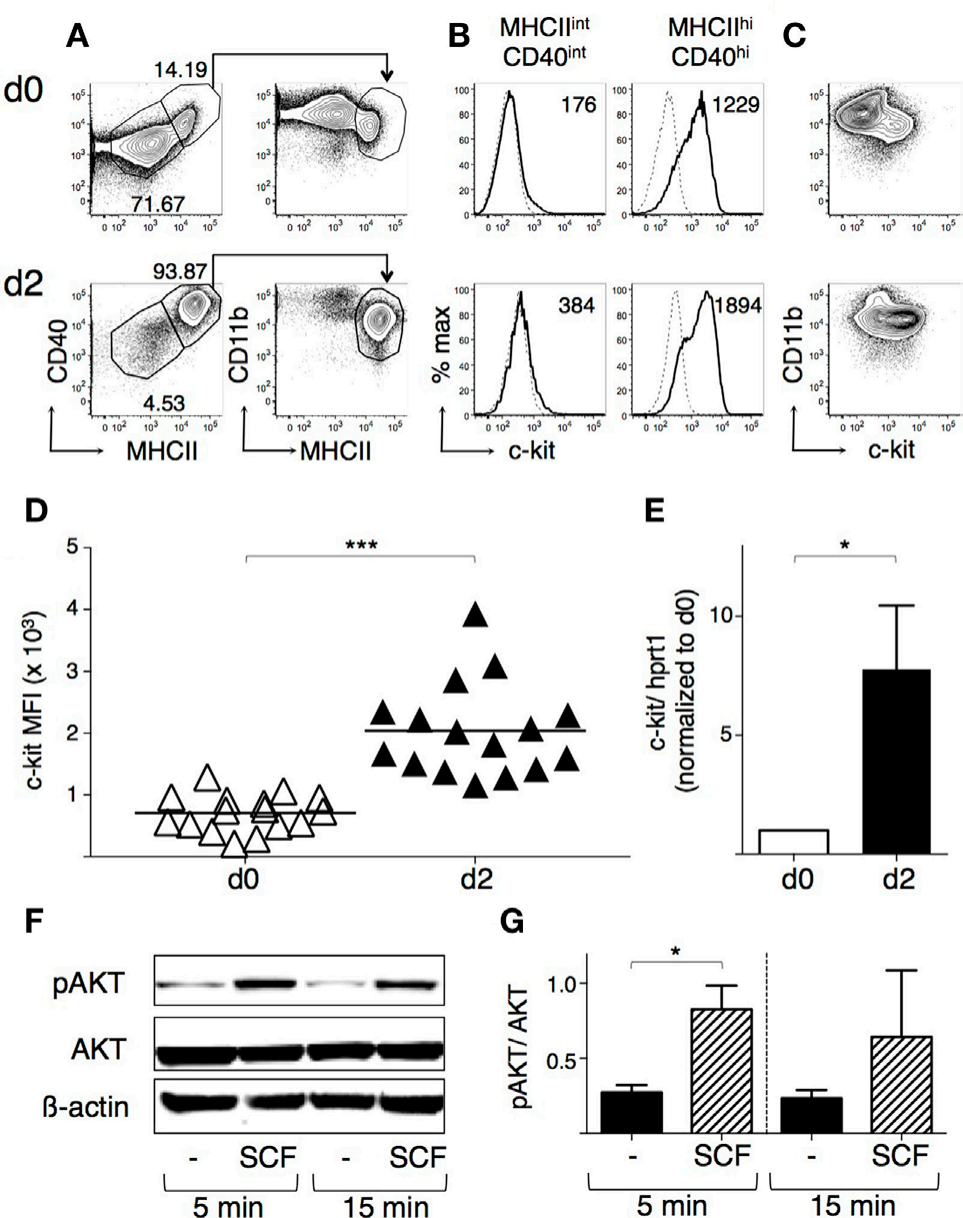
**BM-derived DCs (BMdDCs) express a functional c-kit receptor**. BMdDCs were obtained by purifying CD11c^+^ cells from bone marrow cells cultured with granulocyte-macrophage colony-stimulating factor (GM-CSF) for 1 week, as explained in Section “Materials and Methods” (day 0). BMdDCs were plated in 24-well plates and cultured for 2 days in complete Opti-MEM medium with GM-CSF at 20 ng/ml (day 2). **(A–D)** Analysis of c-kit membrane expression by flow cytometry. Day 0 and day 2 BMdDCs were stained with fluorochrome-conjugated monoclonal antibodies (mAbs) and analyzed by flow cytometry (for gating strategy, see Figure S3 in Supplementary Material). **(A)** Typical flow cytometric profiles showing CD40, CD11b, and MHCII expression by BMdDCs. In the left panels, numbers represent percentages of cells in the indicated regions. **(B)** Typical histograms showing c-kit expression by MHCII^int^ CD40^int^ and MHCII^hi^ CD40^hi^ BMdDCs, gated as in **(A)**. Solid lines represent c-kit staining profiles, dashed lines isotype control mAb. Numbers indicate c-kit median fluorescence intensity (MFI) values. **(C)** Representative contour plots showing c-kit and CD11b expression by BMdDCs. **(D)** Summary of c-kit expression results obtained from day 0 and day 2 MHCII^hi^ CD40^hi^ BMdDCs, gated as in **(A)**. c-kit MFI from individual samples and average values (bar). **(E)** Analysis of c-kit mRNA expression by Real-Time PCR. Day 0 and day 2 BMdDC samples were analyzed by Real-Time PCR in triplicates. c-kit mRNA expression was calculated relative to hprt1 in arbitrary units. For each experiment, day 2 c-kit/hprt1 levels were normalized with day 0. **(F,G)** Western blot analysis of phospho-AKT expression by BMdDCs stimulated with stem cell factor (SCF). Day 2 BMdDCs obtained as above were stimulated with SCF at 100 ng/ml for 5 and 15 min or left untreated, as indicated. Western blot was performed with anti-phospho-AKT, anti-AKT and anti-β actin mAbs, and results analyzed by densitometry. **(F)** Representative Western blot results. **(G)** Densitometric analysis. Phospho-AKT levels were calculated relative to AKT in arbitrary units. In **(A–C)** representative data of 9–16 experiments, in **(D)** *N* = 16 experiments, in **(F)** representative data of three experiments, in **(E,G)** mean ± SD of three experiments (*P ≤ 0.05; ***P ≤ 0.001).

We compared c-kit membrane expression by DCs obtained from mouse BM cells differentiated *in vitro* with either GM-CSF or Flt3-L (40–42). Both protocols resulted in a small percentage of c-kit^+^ within CD11c^+^ cells (Figures S3B,D in Supplementary Material; gating strategy in Figures S3A,C in Supplementary Material). However, DCs generated with GM-CSF were characterized by increased cell yield and higher cell viability (Figures S3A–D in Supplementary Material and data not shown). Thus, we decided to use GM-CSF for further experiments and to magnetically select CD11c^+^ cells from the fraction including non-adherent and slightly adherent cells at day 7 to obtain highly purified DCs (called “BM-derived DCs or BMdDCs,” see Figures S3E,F in Supplementary Material). On average, 15% of the BMdDCs were MHC-II^hi^ CD40^hi^ CD11b^int^ and 76% MHC-II^int^ CD40^int^ CD11b^hi^ cells (see example in Figure 3A, top panel). c-kit was expressed only by the MHC-II^hi^ CD40^hi^ cells, suggesting that it is a marker of the more mature cells (Figure 3B, top panels). The c-kit^+^ BMdDCs were all CD11b^int^, whereas the remaining c-kit^−^ cells were CD11b^hi^ (Figure 3C, top panel).

To increase the proportion of c-kit^+^ cells, we replated BMdDCs in different culture conditions, all with GM-CSF at 20 ng/ml, and then analyzed their membrane phenotype (Figures S4A,B in Supplementary Material and data not shown). After 2 days of culture in complete Opti-MEM medium, the majority of BMdDCs were MHC-II^hi^ CD40^hi^ CD11b^int^, and expressed c-kit^+^ (Figures 3A–C, bottom panels). Membrane c-kit fluorescence intensity significantly increased within the MHC-II^hi^ CD40^hi^ cells, while the MHC-II^int^ CD40^int^ cells remained negative for this marker (Figures 3B,D). c-kit mRNA expression increased significantly, indicating that membrane c-kit up-regulation depended on increased transcription (Figure 3E).

We usually collected non-adherent and slightly adherent BMdDCs when we harvested cells 2 days after replating. In some experiments, we separately analyzed adherent cells and found that they comprised a higher proportion of MHC-II^int^ CD40^int^ cells compared to the floating fraction of BMdDCs. Nevertheless, a high percentage (>70%) of adherent cells was still MHC-II^hi^ CD40^hi^, confirming that MHC-II^hi^ CD40^hi^ cells represented the large majority of BMdDCs after 2 days of culture in our conditions (Figure S4C in Supplementary Material). Notably, as for the non-adherent fraction, the MHC-II^hi^ CD40^hi^ cells were the only c-kit^+^ cells in the adherent fraction (Figure S4D in Supplementary Material).

### SCF/c-Kit Axis Is Functional in BMdDCs and Does Not Influence OVA Presentation by BMdDCs

To determine whether c-kit could function as a signal transducing receptor, c-kit^+^ BMdDCs were stimulated with SCF and analyzed for phosphorylated Akt, a serine-threonine kinase located downstream of PI3-kinase with an essential role in cellular survival and proliferation (34). After incubation with SCF, phospho-Akt levels increased rapidly, while total Akt remained unchanged (Figures 3F,G).

**Figure 4:**
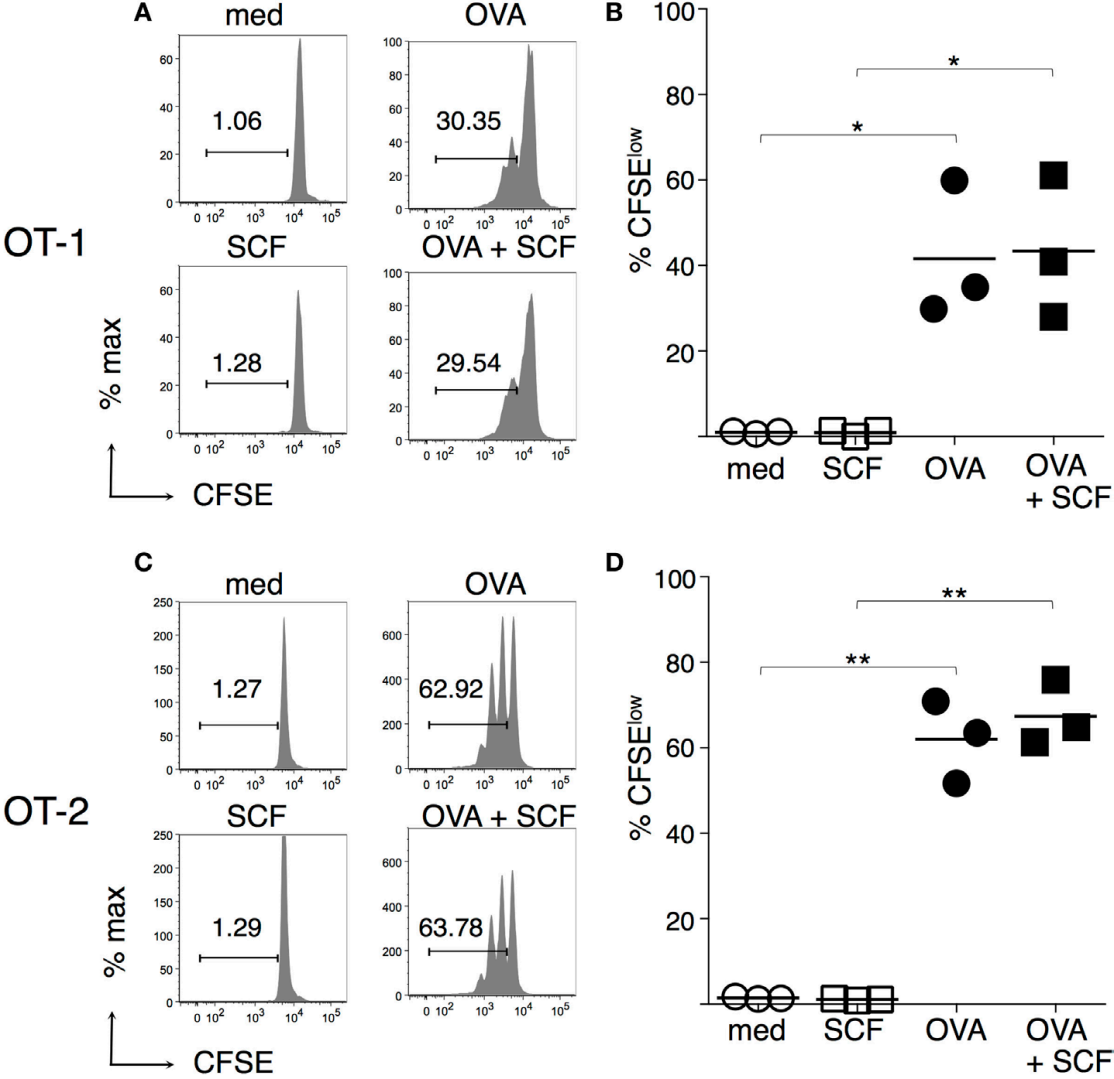
**Antigen presentation by BM-derived DCs (BMdDCs) is not modulated by stem cell factor (SCF)**. Day 2 BMdDCs, obtained as in Figure 3, were incubated for 5 h with OVA at 0.2 mg/ml, in the presence or not of SCF at 100 ng/ml. After extensive washings, BMdDCs were cocultured with either CFSE-labeled purified OT-1 or CFSE-labeled purified OT-2 cells for 3 days. CSFE dilution was evaluated by flow cytometry, **(A,C)** Typical CSFE profiles, after gating on TCR^+^ CD8^+^ for OT-1 **(A)** and TCR^+^ CD4^+^ for OT-2 T cells **(C)**. Numbers represent percentages of cells in the indicated regions. **(B,D)** Summary of OT-1 **(B)** and OT-2 **(D)** proliferation results from individual samples and average values (bar). In **(A,C)** representative data of *N* = 3 experiments, in **(B,D)** *N* = 3 experiments (*P ≤ 0.05; **P ≤ 0.01).

To evaluate the effect of SCF on the capacity of c-kit^+^ BMdDCs to present the model antigen OVA, we assessed the proliferation of OVA-specific T cells from TCR transgenic mice OT-1 and OT-2, by CFSE dilution. We observed that both OT-1 (Figures 4A,B) and OT-2 (Figures 4C,D) cells proliferated in response to BMdDCs preincubated with OVA, showing the functionality of both MHC-I and MHC-II presentation pathways. The presence of SCF during preincubation of BMdDCs with OVA did not influence the proliferation of neither OT-1 (Figures 4A,B) nor OT-2 (Figures 4C,D) cells.

### GM-CSF Inhibits c-Kit Membrane Expression by BMdDCs

**Figure 5:**
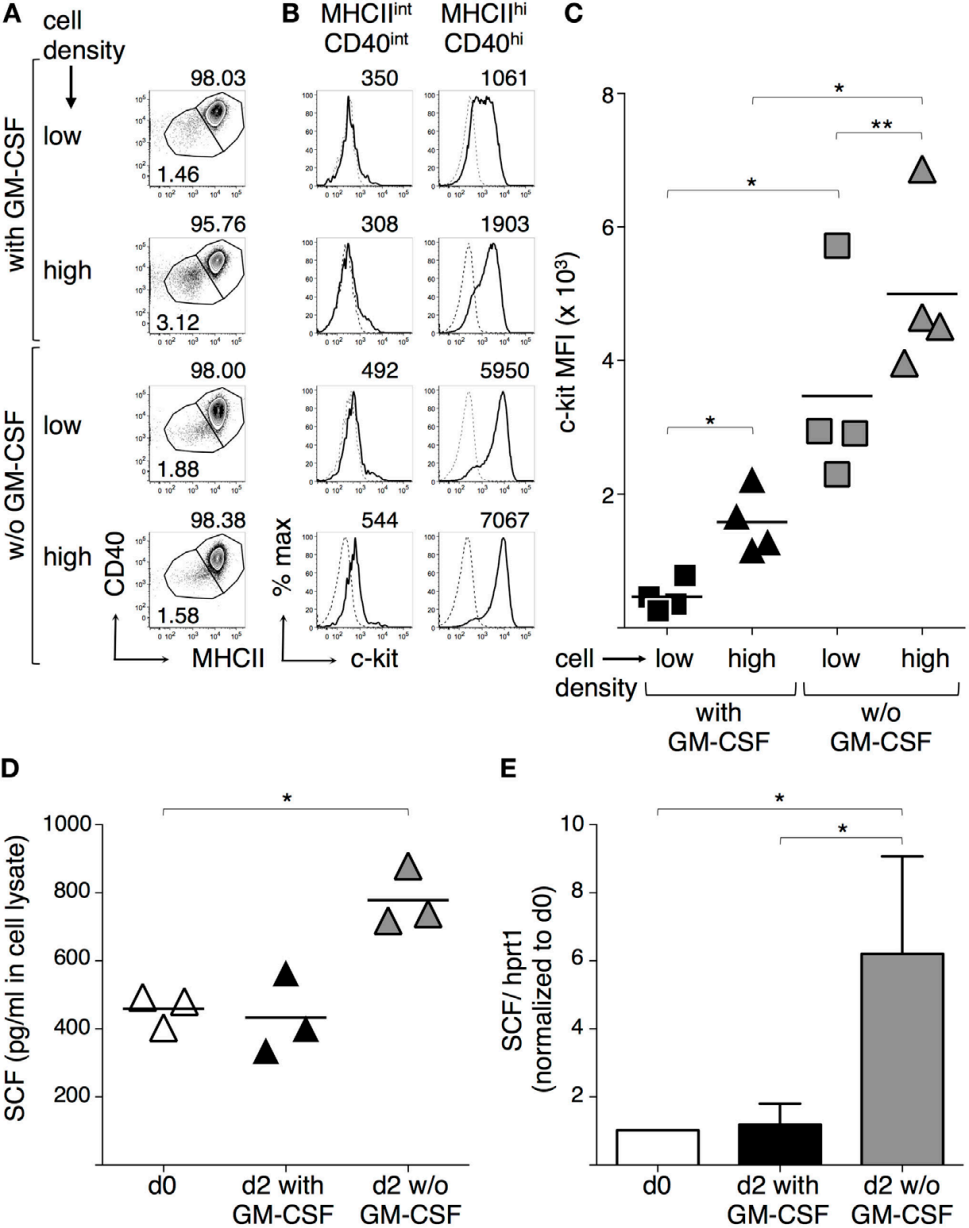
**Granulocyte-macrophage colony-stimulating factor (GM-CSF) modulates c-kit and stem cell factor (SCF) expression by BM-derived DCs (BMdDCs)**. **(A–C)** Effect of GM-CSF and cell density on c-kit expression by BMdDCs. BMdDCs were plated in 24-well plates and cultured for 2 days in complete Opti-MEM medium in four different conditions, that is at either 2 × 10^5^ or 1.2 × 10^5^ cell/well, and either with or without (w/o) GM-CSF at 20 ng/ml, as indicated. Cells were stained with fluorochrome-conjugated monoclonal antibodies (mAbs) and analyzed by flow cytometry. **(A)** Typical flow cytometric profiles, showing CD40 and MHCII expression by BMdDCs. Numbers represent percentages of cells in the indicated regions. **(B)** Typical histograms showing c-kit expression by MHCII^int^ CD40^int^ and MHCII^hi^ CD40^hi^ BMdDCs, gated as in **(A)**. Solid lines represent c-kit staining profiles, dashed lines represent isotype control mAb. Numbers indicate c-kit median fluorescence intensity (MFI) values. **(C)** Summary of c-kit expression results obtained from MHCII^hi^ CD40^hi^ BMdDCs, gated as in **(A)**. c-kit MFI from individual samples and average values (bar). **(D,E)** Effect of GM-CSF on SCF expression by BMdDCs. **(D)** Cell lysates were prepared from day 0 BMdDCs and BMdDCs cultured for 2 days in 24-well plates at 1.2 × 10^5^ cell/well in complete Opti-MEM medium with or w/o GM-CSF at 20 ng/ml, as indicated. SCF protein expression was analyzed by ELISA, testing 25 µg of cell lysate in 100 µl/well. Data are expressed as picograms per milliliter. Individual results from three experiments and average values (bar) are shown. **(E)** Day 0 and day 2 BMdDCs cultured in 24-well plates at 1.2 × 10^5^ cell/well with or w/o GM-CSF at 20 ng/ml were analyzed by Real-Time PCR in triplicates. SCF mRNA expression was calculated relative to hprt1 in arbitrary units. For each experiment, day 2 c-kit/hprt1 levels were normalized with day 0. In **(A,B)** representative data of *N* = 4 experiments, in **(C)** *N* = 4 experiments, in **(D)** *N* = 3 experiments, in **(E)** mean ± SD of four experiments (*P ≤ 0.05; **P ≤ 0.01).

Since GM-CSF can inhibit c-kit expression by mast cells (49, 50), we next evaluated the effects of GM-CSF in our BMdDC cultures, by comparing c-kit membrane expression by BMdDCs cultured in complete Opti-MEM medium either with or without GM-CSF at 20 ng/ml. Furthermore, considering the possible intercellular competition for GM-CSF, we cultured BMdDCs at two different cell densities, i.e., about 6.3 × 10^5^ and 1 × 10^5^ cells/cm^2^, corresponding to 1.2 × 10^6^ and 0.2 × 10^6^ cells/well in 24-well plate, respectively. We observed that c-kit membrane expression by MHC-II^hi^ CD40^hi^ cells was higher when BMdDCs were cultured at higher cell density, and in particular, MHC-II^hi^ CD40^hi^ cells had extremely high c-kit fluorescence intensity when cultured without GM-CSF if compared to BMdDCs cultured in the presence of GM-CSF (Figures 5A–C).

Stem cell factor can be produced by a variety of cell types, including DCs (25, 29, 30). We found that BMdDCs produced cell-associated SCF and its levels remained stable after 2 days of culture in complete Opti-MEM medium with GM-CSF. When BMdDCs were cultured in the absence of GM-CSF, cell-associated SCF significantly increased (Figure 5D). SCF concentration in supernatants was 50.33 ± 1.05 pg/ml after 2 days of culture in the presence of GM-CSF and did not increase in the absence of GM-CSF (data not shown). Notably, SCF expression increased at the mRNA level when BMdDCs were cultured for 2 days without GM-CSF (Figure 5E). Thus, BMdDCs can produce SCF, but the presence of GM-CSF interferes with this process.

### SCF Does Not Modulate CXCR4 Membrane Expression by BMdDCs

**Figure 6:**
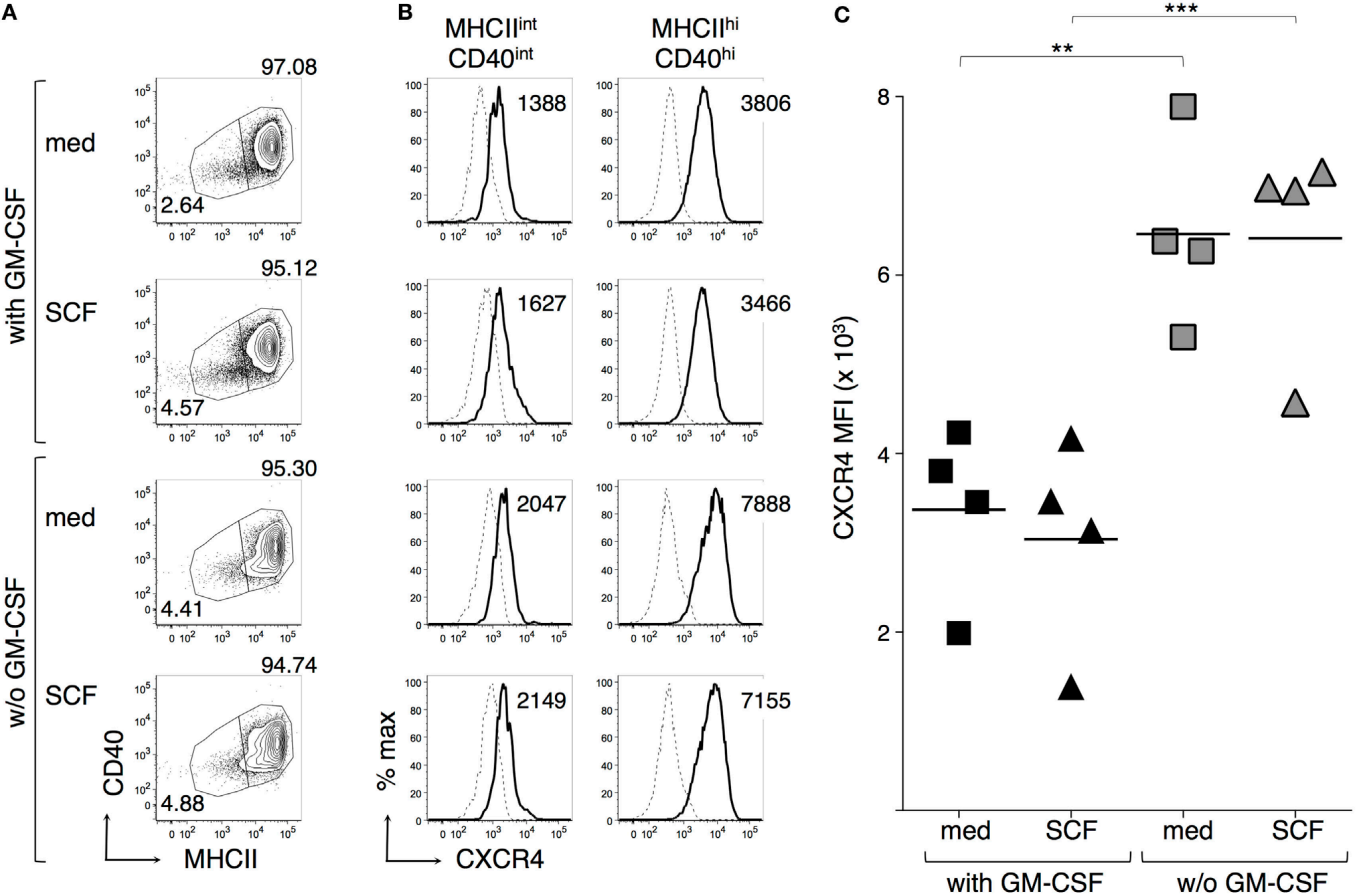
**Granulocyte-macrophage colony-stimulating factor (GM-CSF) but not stem cell factor (SCF) modulates CXCR4 expression by BM-derived DCs (BMdDCs)**. BMdDCs were plated in 24-well plates and cultured for 2 days in complete Opti-MEM medium either with or without (w/o) GM-CSF at 20 ng/ml. SCF was then added at 100 ng/ml and cells were further incubated for 16 h. Cells were stained with fluorochrome-conjugated monoclonal antibodies and analyzed by flow cytometry. **(A)** Typical CD40 and MHCII expression profiles. Numbers represent percentages of cells in the indicated regions. **(B)** Typical histograms showing CXCR4 expression by MHCII^int^ CD40^int^ and MHCII^hi^ CD40^hi^ BMdDCs, gated as in **(A)**. Solid lines represent CXCR4 staining profiles, dashed lines represent no Ab (FMO, Fluorescence Minus One). Numbers indicate CXCR4 median fluorescence intensity (MFI) values. **(C)** Summary of CXCR4 expression results obtained from MHCII^hi^ CD40^hi^ BMdDCs, gated as in **(A)**. CXCR4 MFI from individual samples and average values (bar). In **(A,B)** representative data of *N* = 4 experiments, in **(C)** *N* = 4 experiments (**P ≤ 0.01; ***P ≤ 0.01).

CXCR4 plays a key role in leukocyte retention in the BM, by interacting with CXCL12 (51). Moreover, DC maturation results in upregulation of CXCR4 (3, 5). We compared CXCR4 membrane expression by BMdDCs cultured in complete Opti-MEM medium either with or without GM-CSF at 20 ng/ml and evaluated whether an overnight addition of SCF to these cultures modulated CXCR4. MHC-II^hi^ CD40^hi^ cells had always a higher CXCR4 MFI than MHC-II^int^ CD40^int^ cells (Figures 6A,B). CXCR4 expression by MHC-II^hi^ CD40^hi^ cells increased in the absence of GM-CSF, and the addition of SCF to the culture did not influence the expression of CXCR4 (Figures 6B,C).

### SCF Is an Autocrine Survival Factor for BMdDCs

**Figure 7:**
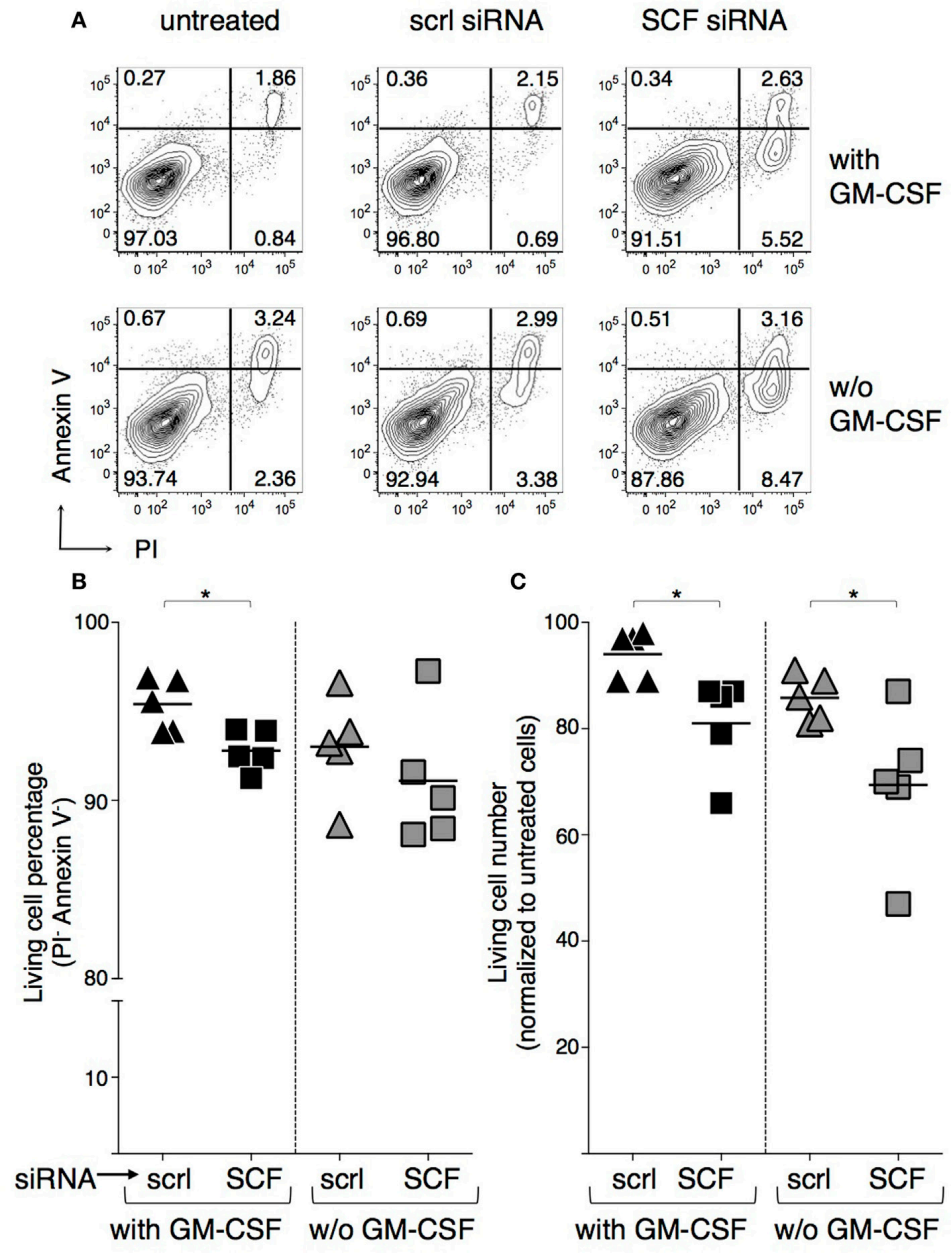
**Negative effect of stem cell factor (SCF) silencing on BM-derived DC (BMdDC) survival**. BMdDCs were transfected with either SCF-siRNA or control-scrambled (scrl) siRNA, or else left untreated. Cells cultured in triplicates in 96-well plates at 2 × 10^5^/well in complete Opti-MEM medium with or w/o granulocyte-macrophage colony-stimulating factor (GM-CSF) at 20 ng/ml were analyzed after 2 days. **(A,B)** Percentages of living cells. Flow cytometry analysis was performed after staining with Annexin V FITC and incubation with PI. **(A)** Typical Annexin V and PI staining profiles. Numbers represent percentages of cells in the indicated quadrants. Living cells are in the lower left quadrant (Annexin V^−^ PI^−^). **(B)** Summary of results obtained by analyzing living cell percentages among SCF-siRNA and control-scrambled siRNA treated BMdDCs, gated as in **(A)**. Percentages of living cells from individual samples and average values (bar). **(C)** Numbers of living cells. BMdDC numbers were evaluated by the CyQuant assay. Results of SCF-siRNA and control-scrambled siRNA treated samples were normalized over corresponding untreated BMdDCs. Numbers of living cells from individual samples and average values (bar). In **(A)** representative data of *N* = 5 experiments, in **(B,C)** *N* = 5 experiments (*P ≤ 0.05).

To evaluate whether BMdDC survival could be modulated by SCF autocrine production, SCF was silenced by transfection with SCF-siRNA and cell survival analyzed after 2 days of culture either with or without GM-CSF. In our conditions, transfection efficiency was about 40%, as evaluated in control cells transfected in parallel with fluorescein-conjugated RNA, and SCF protein concentration in lysates from SCF-siRNA-silenced cells was about 70% than that in lysates from scrambled siRNA-silenced control cells (e.g., 489 pg/ml in SCF-silenced cells and 671 pg/ml in control cells, in cultures with GM-CSF; 589 pg/ml in SCF-silenced cells and 714 pg/ml in control cells, without GM-CSF). Although SCF expression was only partially reduced, we observed a statistically significant decrease in cell survival in the cultures with GM-CSF, based on Annexin V/PI staining (Figures 7A,B). Absolute cell numbers were significantly decreased upon SCF silencing in both BMdDC cultures, with and without GM-CSF (Figure 7C). In a different set of experiments, we cultured BMdDCs for 2 days in the presence of 10 µg/ml of either the anti-c-kit blocking mAb ACK2 or its corresponding control mAb and observed a modest but statistically significant decrease in cell survival, based on Annexin V/PI staining (Figure S5 in Supplementary Material). Results were similar in BMdDC culture with and without GM-CSF (Figure S5 in Supplementary Material). Altogether, these findings indicate that DCs promote their own survival both by the autocrine production of SCF and by expressing the receptor c-kit.

### c-Kit Is Preferentially Expressed by cDC1s in Human BM

**Figure 8:**
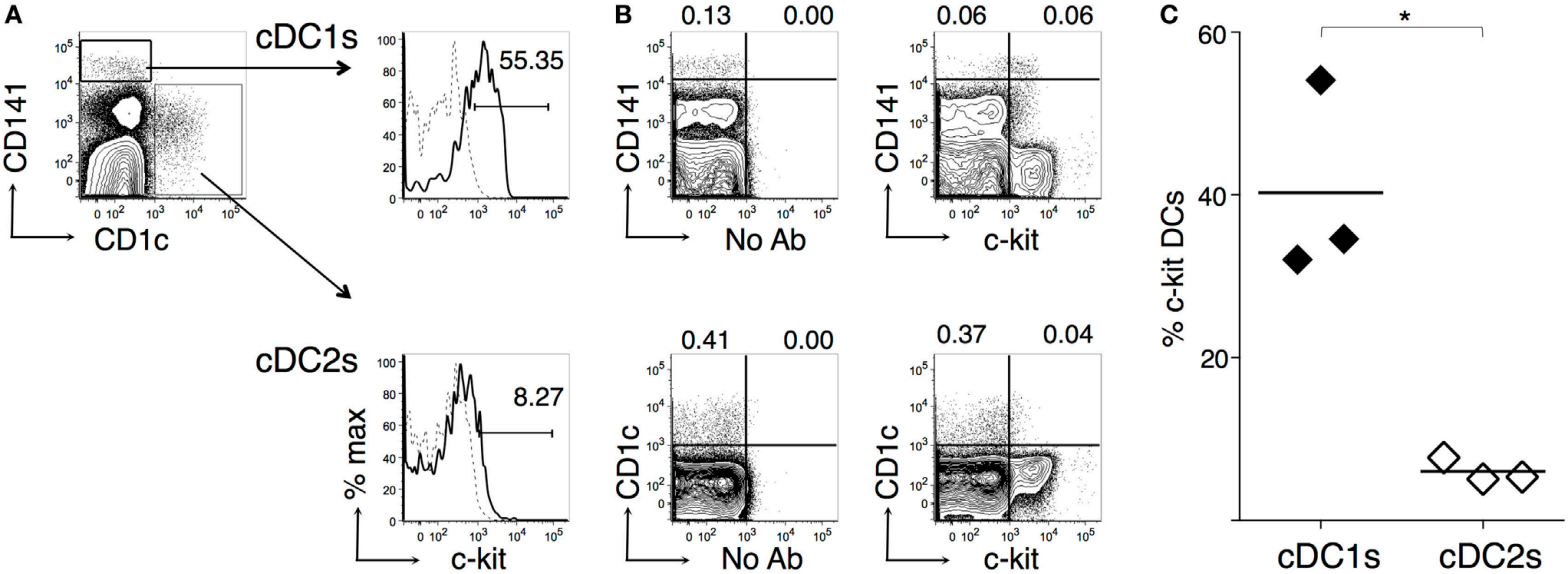
**c-Kit membrane expression by human bone marrow (BM) type 1 cDC subsets (cDC1s) and type 2 cDC subsets (cDC2s)**. Single-cell suspensions were prepared from human BM samples obtained from patients undergoing orthopedic surgery. After density gradient, cells were stained with fluorochrome-conjugated monoclonal antibodies (mAbs) and analyzed by flow cytometry (for gating strategy, see Figure S1B in Supplementary Material). **(A)** Typical flow cytometric profiles, showing c-kit^+^ cell percentages among CD141^hi^ cDC1s and CD1c^+^ cDC2s. In histograms, solid lines represent c-kit staining profiles, dashed lines represent no Ab (FMO). The numbers represent percentages of cells in the indicated regions. **(B)** Representative contour plots showing c-kit, CD141, and CD1c expression by BM cells. The numbers represent percentages of cells in the indicated regions. **(C)** Summary of results obtained from BM of individual patients and average values of each group (bar). Percentages of c-kit^+^ cells were obtained after subtracting FMO background values. In **(A,B)** representative data of *N* = 3 in three experiments, in **(C)** *N* = 3 in three experiments (*P ≤ 0.05).

We also analyzed cDC from human BM samples, by using high levels of CD141 and expression of CD1c as markers for cDC1s and cDC2s, respectively (14, 52). Typical examples of human BM cytometric profiles are shown in Figures 8A,B (gating strategy in Figure S1B in Supplementary Material). In agreement with mouse data, we found that cDC1s contained a significantly higher percentage of c-kit^+^ cells than cDC2s (Figure 8C). c-Kit histograms suggested that cDC1s represented a homogeneous subset expressing c-kit at low levels, rather than a heterogeneous population containing a negative and a positive fraction (Figure 8A). Further analysis showed that the CD141^−^ fraction in the BM contained cells expressing much higher c-kit levels than cDC1s (Figure 8B).

## Discussion

Our data show that c-kit^+^ DCs are found in human BM, and mouse spleen and BM. BM c-kit^+^ DCs had intermediate expression of GFP in the CX3cr1^gfp/+^ reporter mouse, distinguishing them from the previously described perivascular BM-resident DC subset (47). By comparing the two cDC subsets in the BM, we found that both human and mouse BM cDC1s were homogeneous cell populations, expressing low and intermediate levels of c-kit, respectively, whereas cDC2s were heterogeneous and differed between humans and mice. c-Kit was expressed by a fraction of mouse BM cDC2s, while human BM cDC2s had little or no expression of c-kit, thus echoing human blood cDC2s (53). The difference in c-kit expression between BM cDC1s and cDC2s is not surprising, in light of the distinct developmental pathways and composition of these two subsets. Indeed, cDC1s are more homogeneous and their development strongly requires the transcription factor BATF3, while cDC2s mostly depend on IRF4 and comprise various subpopulations (2, 14). In further support of cDC2 heterogeneity, we found that—differently from mouse BM cDC2s—mouse spleen cDC2s were virtually all c-kit^+^.

c-kit^+^ DCs represented a small percentage of DCs differentiated from mouse BM cells *in vitro* with either Flt3-L or GM-CSF. It is known that DCs differentiated from BM cells with GM-CSF are heterogeneous for the expression of typical activation markers, e.g., MHC-II, CD40 (54, 55). We followed a widely used protocol to generate DCs (41), and after purification of CD11c^+^ cells at day 7, we obtained a mixture of c-kit^−^ CD40^int^ MHC-II^int^ and c-kit^+^ CD40^hi^ MHC-II^hi^ cells. However, after replating these BMdDCs in complete Opti-MEM medium with GM-CSF for 2 days, we obtained a quite homogeneous set of c-kit^+^ CD40^hi^ MHC-II^hi^ cells, which expressed a functional c-kit receptor, as demonstrated by Akt phosphorylation after a short incubation with SCF. Upon incubation with the model antigen OVA, these cells were able to present OVA-derived peptides to both OT-1 and OT-2 cells, showing that both MHC-I and MHC-II antigen presentation pathways were functional. Results were similar with or without SCF. We also investigated whether CXCR4 expression by BMdDCs could be modulated by GM-CSF and/or SCF and observed that GM-CSF negatively regulated CXCR4 membrane expression, while SCF did not have any effect. This suggests that SCF does not regulate retention of DCs in the BM, while GM-CSF might promote DC mobilization out of the BM, by reducing CXCR4 expression. GM-CSF-mediated inhibition of CXCR4 expression by DCs resembles GM-CSF effect on neutrophils (56).

We documented a negative effect of GM-CSF on both c-kit expression and SCF production by BMdDCs. Indeed, c-kit expression strikingly increased if GM-CSF was omitted after replating BMdDCs in complete Opti-MEM. We also observed that c-kit expression significantly increased at high cell density with GM-CSF at 20 ng/ml, possibly reflecting reduced availability of GM-CSF due to intercellular competition in the culture. The fact that, even without adding GM-CSF, high-density BMdDC cultures resulted in increased c-kit expression suggests that further mechanisms regulate c-kit expression, for example, changes in pH, or competition for nutrients in the medium. Furthermore, we showed that BMdDCs produced SCF and that both SCF mRNA and protein augmented when cells were cultured without GM-CSF for 2 days after replating. In particular, cell-associated SCF increased, but not SCF found in the culture supernatant, suggesting either that membrane-bound SCF was the prevalent isoform or that soluble SCF was consumed in the culture.

The inverse correlation between addition of GM-CSF to the culture medium and expression of c-kit/SCF by BMdDCs prompted us to investigate whether autocrine SCF production could play a role in BMdDC survival. In this regard, we found that SCF silencing by siRNA resulted in reduced BMdDC survival, suggesting that SCF could contribute to DC maintenance. The prosurvival role of SCF in DC survival has not been previously recognized, possibly due to the inhibitory effects of GM-CSF on SCF/c-kit axis. Indeed, it should not be overlooked that GM-CSF is widely employed to culture mouse and human DCs *in vitro*, including human DCs used in clinical settings (57–59). Based on our study, alternative methods to culture differentiated DCs *in vitro* might be developed, possibly exploiting SCF/c-kit circuit (60–62).

We speculate that DCs more prominently rely on SCF *in vivo* in some microenvironments, for example, in conditions of low GM-CSF and high SCF concentration, that might be found in some BM niches. To obtain evidence of SCF/c-kit function in differentiated DCs *in vivo*, it would be necessary to develop new models of genetically manipulated mice, as available mutant mice have major developmental defects. Indeed, genetic deficiencies in either c-kit or SCF are often lethal in mice, or can lead to severe defects of hematopoiesis, melanocyte, and germ cell development (63, 64), making it difficult to examine DCs. In case of hypomorphic c-kit mutations, myeloid lineage defects were not usually reported (65), nevertheless the absolute number of CMPs was significantly decreased in mice bearing the W^41^ hypomorphic mutation (66), suggesting an abnormal development of CMP-derived cells, including DCs. Notably, c-kit is expressed not only by CMPs, but also by common DC precursors in the BM (24, 67, 68). Thus, novel tools have to be developed to specifically investigate the role of SCF/c-kit in differentiated DC survival, for example, DC-specific c-kit conditional ko mice.

Our results on c-kit^+^ BM DCs might be relevant for some hematological diseases in which immune responses occur in the BM, such as graft-versus-host disease (GVHD) and graft-versus-leukemia (69–71). It is now recognized that the BM niche for HSC is one of the major targets of GVHD, in addition to gut, liver, and skin (70–72). In this context, local DCs in the BM can orchestrate T cell activation (73), thus regulating target organ damage. Based on our results, it might be speculated that changes in BM SCF levels occurring in BM transplantation (74, 75) can affect DC survival, possibly influencing several steps of local and systemic immune response. Moreover, a reduced expression of either SCF or c-kit might play a role in BM DC survival in some diseases of unclear pathogenesis, for example high-risk myelodysplastic syndromes (76), and sepsis-induced immunosuppression (77, 78).

Notably, c-kit^+^ DCs have been implicated in the immune response against cancer, but their role is still unclear. On one hand, it was proposed that some indirect anticancer effects of therapy with imatinib mesylate might be due to drug-mediated inhibition of c-kit and/or other tyrosine kinase receptors expressed by DCs (79). On the other hand, microarray analysis of intratumoral DCs from cancers with different prognosis intriguingly demonstrated that the top gene positively correlated with favorable prognosis is c-kit (80). Thus, further studies are required to determine the role of c-kit^+^ DC, including BM cDC1s, in orchestrating either spontaneous or treatment-induced antitumor immunity.

## Ethics Statement

This study was carried out in accordance with the recommendations of Institutional Ethics Committee of Centro Traumatologico Ortopedico Andrea Alesini Hospital (ASL Roma C) with written informed consent from all subjects. All subjects gave written informed consent in accordance with the Declaration of Helsinki. Approved protocol: study no. 129.14, prot 76699. This study was carried out in accordance with the recommendations of Institutional Ethics Committee of Policlinico Tor Vergata (Fondazione PTV Policlinico Tor Vergata) with written informed consent from all subjects. All subjects gave written informed consent in accordance with the Declaration of Helsinki. Approved protocol: study no. 156.15, prot. 0030053/2015. For mouse experiments, this study was carried out in accordance with the recommendations of institutional guidelines of Istituto Superiore di Sanità of Rome (ISS), DL116/92 and 26/2014.

## Author Contributions

FD designed experiments, interpreted the results, and wrote the paper with help by SS; SV and AQ performed experiments with WT mouse BM and spleen DCs; SV performed experiments with Cx3cr1-mutant mice and WT cDC subsets; ASeijas and SS performed experiments with mouse BMdDCs; ASeijas performed SCF silencing and SCF Elisa experiments; SS analyzed CXCR4, tested SCF effect on antigen presentation, and performed experiments with human BM DCs; FD and SS analyzed flow cytometry data; DR, ASeijas, and ASoriani performed/analyzed Real-Time PCR experiments; MC, IF, and AN performed/analyzed Western Blot experiments; FS, UT, FO, and EP took care of the patients and provided human BM samples; ASantoni provided important conceptual insights.

## Conflict of Interest Statement

The authors declare that the research was conducted in the absence of any commercial or financial relationships that could be construed as a potential conflict of interest.

## Supplementary Materials

Figure S1 **Gating strategy for mouse and human dendritic cells (DCs)**. Cells were stained with fluorochrome-conjugated monoclonal antibodies and analyzed by flow cytometry. **(A)** Mouse bone marrow (BM) and spleen DCs. Gating strategy based on forward and side scatter is shown. In the dump channel, CD3^+^, NK1.1^+^, and PI^+^ cells were excluded. **(B)** Human BM DCs. Gating strategy based on forward and side scatter is shown. The CD45^+^ cells were gated, while CD14^+^, CD19^+^, and dead cell discriminator (DCD)^+^ cells were excluded in the dump channel.

Figure S2 **c-Kit membrane expression by dendritic cells (DCs) from WT and Cx3cr1-genetically modified mice**. **(A,B)** CD11c^+^ cells were enriched from WT bone marrow (BM) and spleen by immunomagnetic selection. **(C,D)** BM and spleen cells were obtained from Cx3cr1^gfp/+^, Cx3cr1^gfp/gfp^, and WT mice. Cells were stained with fluorochrome-conjugated monoclonal antibodies (mAbs) and analyzed by flow cytometry. Typical flow cytometric profiles, showing c-kit^+^ cell percentages among CD11c^high^ MHCII^+^ DCs from BM **(A,C)** and spleen **(B,D)**. In the histograms, solid lines represent c-kit staining profiles, dashed lines isotype control mAb. Numbers represent percentages of cells in the indicated regions. In **(A,B)** representative data from *N* = 5 experiments, in **(C,D)** representative data from *N* = 9 in three experiments (1 mouse/group in each experiment).

Figure S3 **Gating strategy for dendritic cells (DCs) generated *in vitro* from mouse bone marrow (BM)**. Cells were stained with fluorochrome-conjugated monoclonal antibodies (mAbs) and analyzed by flow cytometry. Gating strategy based on forward/side scatter and dead cell exclusion by PI is shown for DCs generated from BM cells with FMS-like tyrosine kinase 3 ligand (Flt3-L) **(A)** and with granulocyte-macrophage colony-stimulating factor (GM-CSF) **(C,E)**. c-kit expression is shown for DCs generated with Flt3-L **(B)** and with GM-CSF **(D,F)**. Panels **(E,F)** show results obtained with GM-CSF after cell purification with anti-CD11c magnetic microbeads. Histograms show results obtained with CD11c^+^ cells, gated as shown; solid lines represent c-kit staining profiles, dashed lines indicate isotype control mAb.

Figure S4 **c-Kit expression by BM-derived DCs (BMdDCs): comparison of different culture media and analysis of adherent and non-adherent cells**. **(A,B)** Culture media. BMdDCs were plated in 24-well plates and cultured for 2 days with granulocyte-macrophage colony-stimulating factor (GM-CSF) at 20 ng/ml either in complete RPMI medium, or in complete Opti-MEM medium. Complete RPMI medium contains 10% fetal calf serum (FCS); complete Opti-MEM medium is serum free (see Section “Materials and Methods” for details). Cells were stained with fluorochrome-conjugated monoclonal antibodies (mAbs) and analyzed by flow cytometry, as in Figure 3. **(A)** Typical flow cytometric profiles, showing CD40 and MHCII expression by BMdDCs. Numbers represent percentages of cells in the indicated regions. **(B)** Typical histograms showing c-kit expression by MHCII^int^ CD40^int^ and MHCII^hi^ CD40^hi^ BMdDCs, gated as in **(A)**. Solid lines represent c-kit staining profiles, dashed lines indicate isotype control mAb. Numbers indicate c-kit median fluorescence intensity values. **(C,D)** Adherent and non-adherent cells. BMdDCs were plated in 24-well plates and cultured for 2 days in complete Opti-MEM medium with GM-CSF at 20 ng/ml, before harvesting either non-adherent cells or adherent cells after detachment with PBS 10 mM EDTA. Cells were analyzed and results represented as in **(A,B)**. In **(A,B)** representative data from *N* = 3 experiments; in **(C,D)** representative data from *N* = 5 experiments.

Figure S5 **Negative effect of anti-c-kit blocking monoclonal antibody (mAb) on BM-derived DC (BMdDC) survival**. BMdDCs were cultured for 2 days in triplicates in 96-well plates at 2 × 10^5^/well in complete Opti-MEM medium in four different conditions, that is either with or without (w/o) granulocyte-macrophage colony-stimulating factor (GM-CSF), and in the presence of 10 µg/ml of either the anti-c-kit blocking mAb ACK2 or its isotype control (ctrl) mAb, as indicated. Flow cytometry analysis was performed after staining with Annexin V FITC and incubation with PI. **(A)** Typical Annexin V and PI staining profiles. Numbers represent percentages of cells in the corresponding quadrants. Living cells are in the lower left quadrant (Annexin V^−^ PI^−^). **(B)** Summary of results. Percentages of living cells from individual samples treated either with anti-c-kit or control mAb. In **(A)** representative data of *N* = 4 experiments, in **(B)** *N* = 4 experiments (**P* ≤ 0.05).
