# Supplementary figures and images for: GM-CSF Inhibits c-Kit and SCF Expression by Bone Marrow-Derived Dendritic Cells
Source: Front Immunol. 2017 Feb 16;8:147. doi: 10.3389/fimmu.2017.00147 (PMC5311071; doi:10.3389/fimmu.2017.00147)

**A****BM**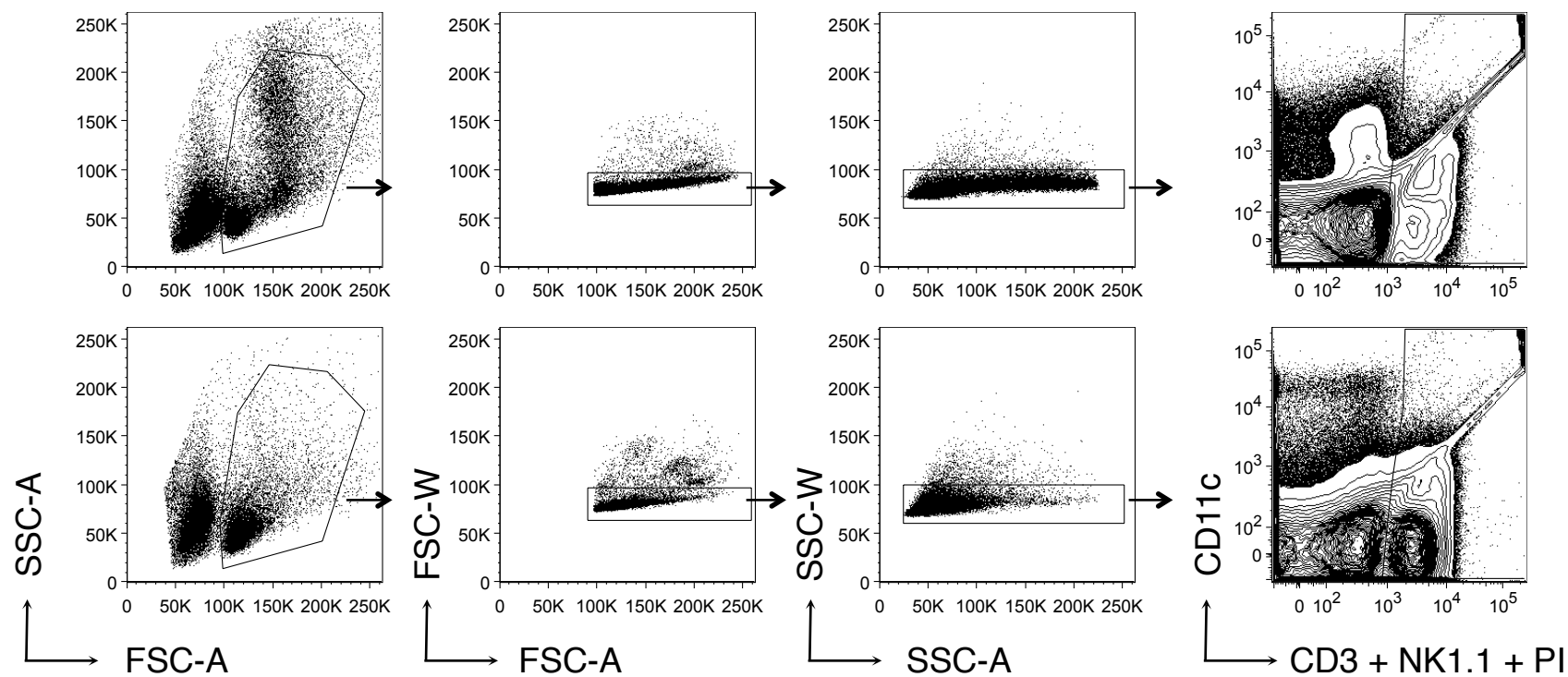**Spl**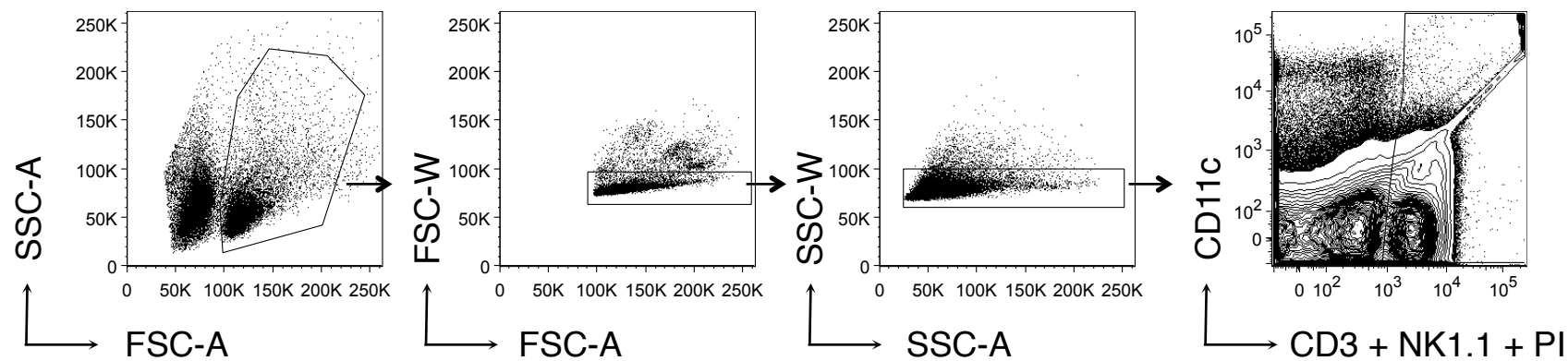**B****Human BM**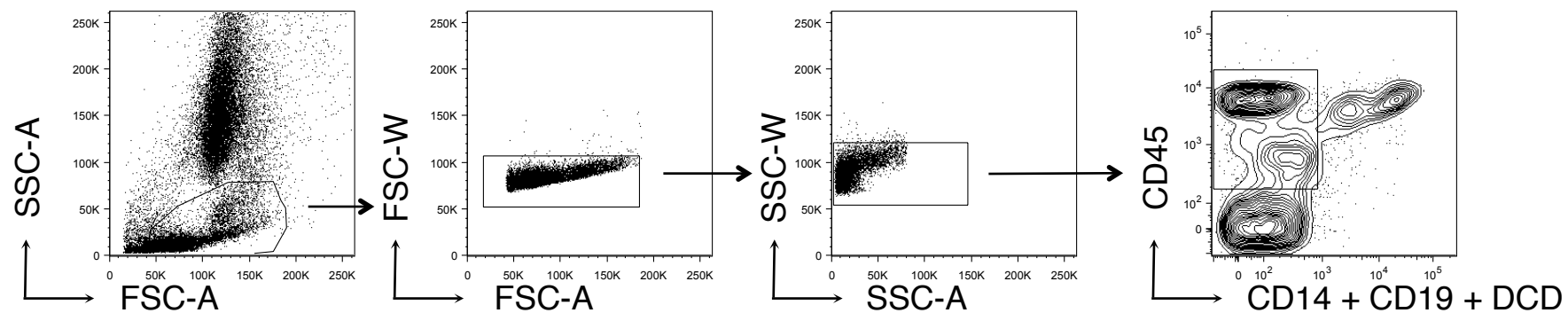**Fig. S1**

Supplement: Figure S1 — Gating strategy for mouse and human dendritic cells (DCs). Cells were stained with fluorochrome-conjugated monoclonal antibodies and analyzed by flow cytometry. (A) Mouse bone marrow (BM) and spleen DCs. Gating strategy based on forward and side scatter is shown. In the dump channel, CD3+, NK1.1+, and PI+ cells were excluded. (B) Human BM DCs. Gating strategy based on forward and side scatter is shown. The CD45+ cells were gated, while CD14+, CD19+, and dead cell discriminator (DCD)+ cells were excluded in the dump channel. [file Image_1.PDF]

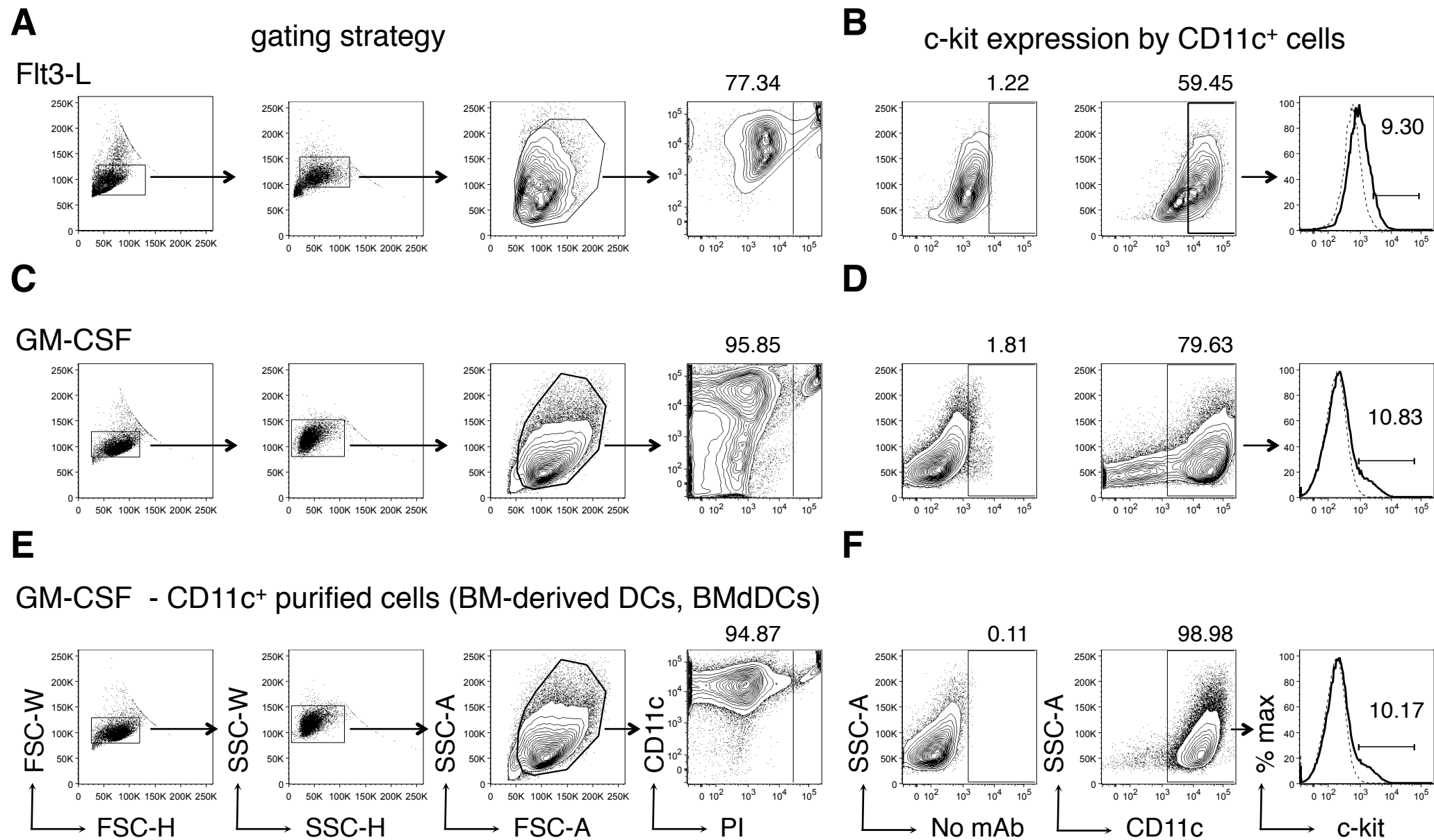

**Fig. S3**

Supplement: Figure S3 — Gating strategy for dendritic cells (DCs) generated in vitro from mouse bone marrow (BM). Cells were stained with fluorochrome-conjugated monoclonal antibodies (mAbs) and analyzed by flow cytometry. Gating strategy based on forward/side scatter and dead cell exclusion by PI is shown for DCs generated from BM cells with FMS-like tyrosine kinase 3 ligand (Flt3-L) (A) and with granulocyte-macrophage colony-stimulating factor (GM-CSF) (C,E). c-kit expression is shown for DCs generated with Flt3-L (B) and with GM-CSF (D,F). Panels (E,F) show results obtained with GM-CSF after cell purification with anti-CD11c magnetic microbeads. Histograms show results obtained with CD11c+ cells, gated as shown; solid lines represent c-kit staining profiles, dashed lines indicate isotype control mAb. [file Image_3.PDF]

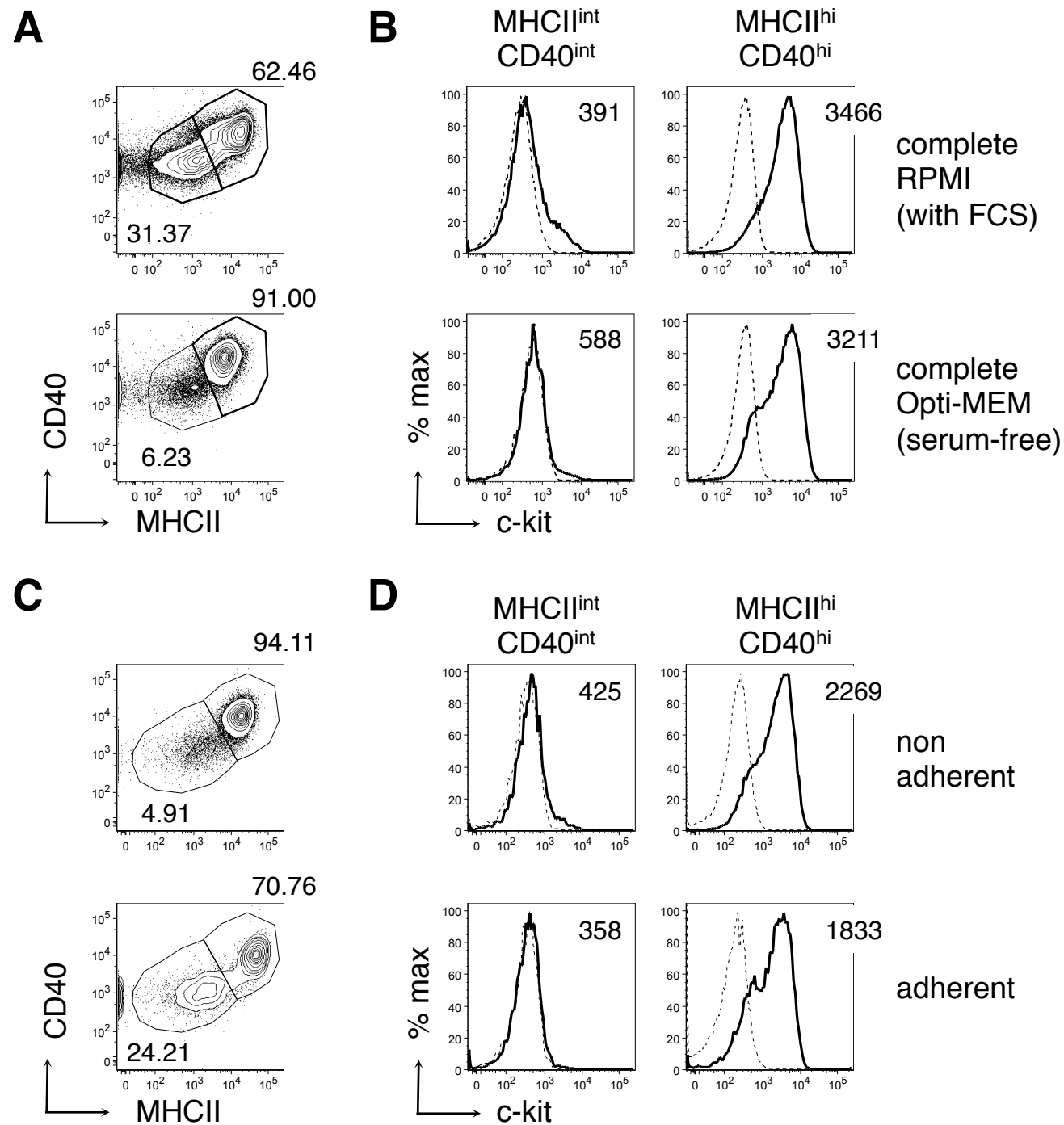

**Fig. S4**

Supplement: Figure S4 — c-Kit expression by BM-derived DCs (BMdDCs): comparison of different culture media and analysis of adherent and non-adherent cells. (A,B) Culture media. BMdDCs were plated in 24-well plates and cultured for 2 days with granulocyte-macrophage colony-stimulating factor (GM-CSF) at 20 ng/ml either in complete RPMI medium, or in complete Opti-MEM medium. Complete RPMI medium contains 10% fetal calf serum (FCS); complete Opti-MEM medium is serum free (see Section “Materials and Methods” for details). Cells were stained with fluorochrome-conjugated monoclonal antibodies (mAbs) and analyzed by flow cytometry, as in Figure 3. (A) Typical flow cytometric profiles, showing CD40 and MHCII expression by BMdDCs. Numbers represent percentages of cells in the indicated regions. (B) Typical histograms showing c-kit expression by MHCIIint CD40int and MHCIIhi CD40hi BMdDCs, gated as in (A). Solid lines represent c-kit staining profiles, dashed lines indicate isotype control mAb. Numbers indicate c-kit median fluorescence intensity values. (C,D) Adherent and non-adherent cells. BMdDCs were plated in 24-well plates and cultured for 2 days in complete Opti-MEM medium with GM-CSF at 20 ng/ml, before harvesting either non-adherent cells or adherent cells after detachment with PBS 10 mM EDTA. Cells were analyzed and results represented as in (A,B). In (A,B) representative data from N = 3 experiments; in (C,D) representative data from N = 5 experiments. [file Image_4.PDF]

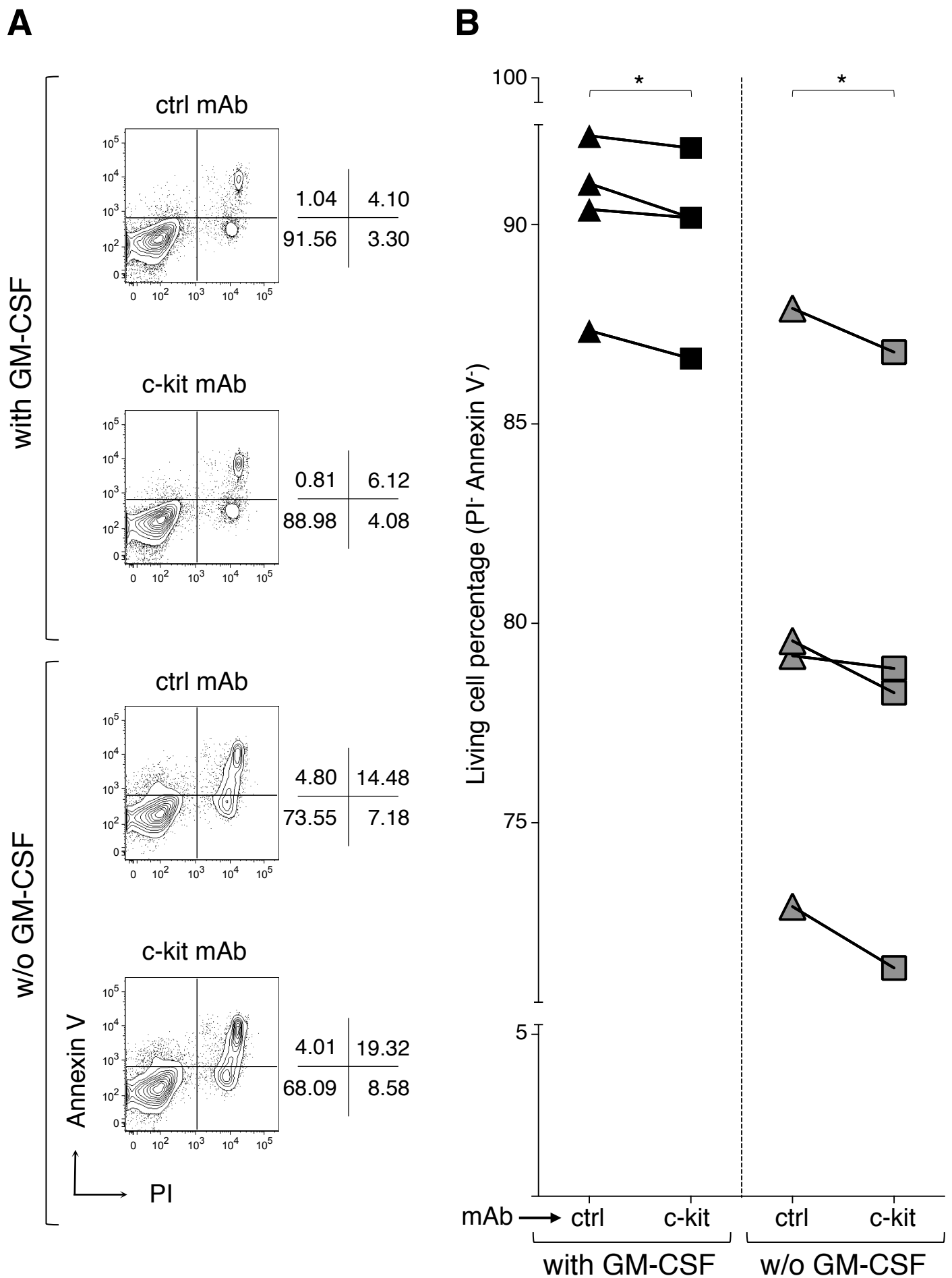

**Fig. S5**

Supplement: Figure S5 — Negative effect of anti-c-kit blocking monoclonal antibody (mAb) on BM-derived DC (BMdDC) survival. BMdDCs were cultured for 2 days in triplicates in 96-well plates at 2 × 10^5^/well in complete Opti-MEM medium in four different conditions, that is either with or without (w/o) granulocyte-macrophage colony-stimulating factor (GM-CSF), and in the presence of 10 µg/ml of either the anti-c-kit blocking mAb ACK2 or its isotype control (ctrl) mAb, as indicated. Flow cytometry analysis was performed after staining with Annexin V FITC and incubation with PI. (A) Typical Annexin V and PI staining profiles. Numbers represent percentages of cells in the corresponding quadrants. Living cells are in the lower left quadrant (Annexin V− PI−). (B) Summary of results. Percentages of living cells from individual samples treated either with anti-c-kit or control mAb. In (A) representative data of N = 4 experiments, in (B) N = 4 experiments (*P ≤ 0.05). [file Image_5.PDF]
