# Supplementary material for: GM-CSF Inhibits c-Kit and SCF Expression by Bone Marrow-Derived Dendritic Cells
Source: Front Immunol. 2017 Feb 16;8:147. doi: 10.3389/fimmu.2017.00147 (PMC5311071; doi:10.3389/fimmu.2017.00147)
Supplement: Figure S2 — c-Kit membrane expression by dendritic cells (DCs) from WT and Cx3cr1-genetically modified mice. (A,B) CD11c+ cells were enriched from WT bone marrow (BM) and spleen by immunomagnetic selection. (C,D) BM and spleen cells were obtained from Cx3cr1gfp/+, Cx3cr1gfp/gfp, and WT mice. Cells were stained with fluorochrome-conjugated monoclonal antibodies (mAbs) and analyzed by flow cytometry. Typical flow cytometric profiles, showing c-kit+ cell percentages among CD11chigh MHCII+ DCs from BM (A,C) and spleen (B,D). In the histograms, solid lines represent c-kit staining profiles, dashed lines isotype control mAb. Numbers represent percentages of cells in the indicated regions. In (A,B) representative data from N = 5 experiments, in (C,D) representative data from N = 9 in three experiments (1 mouse/group in each experiment). [file Image_2.PDF]

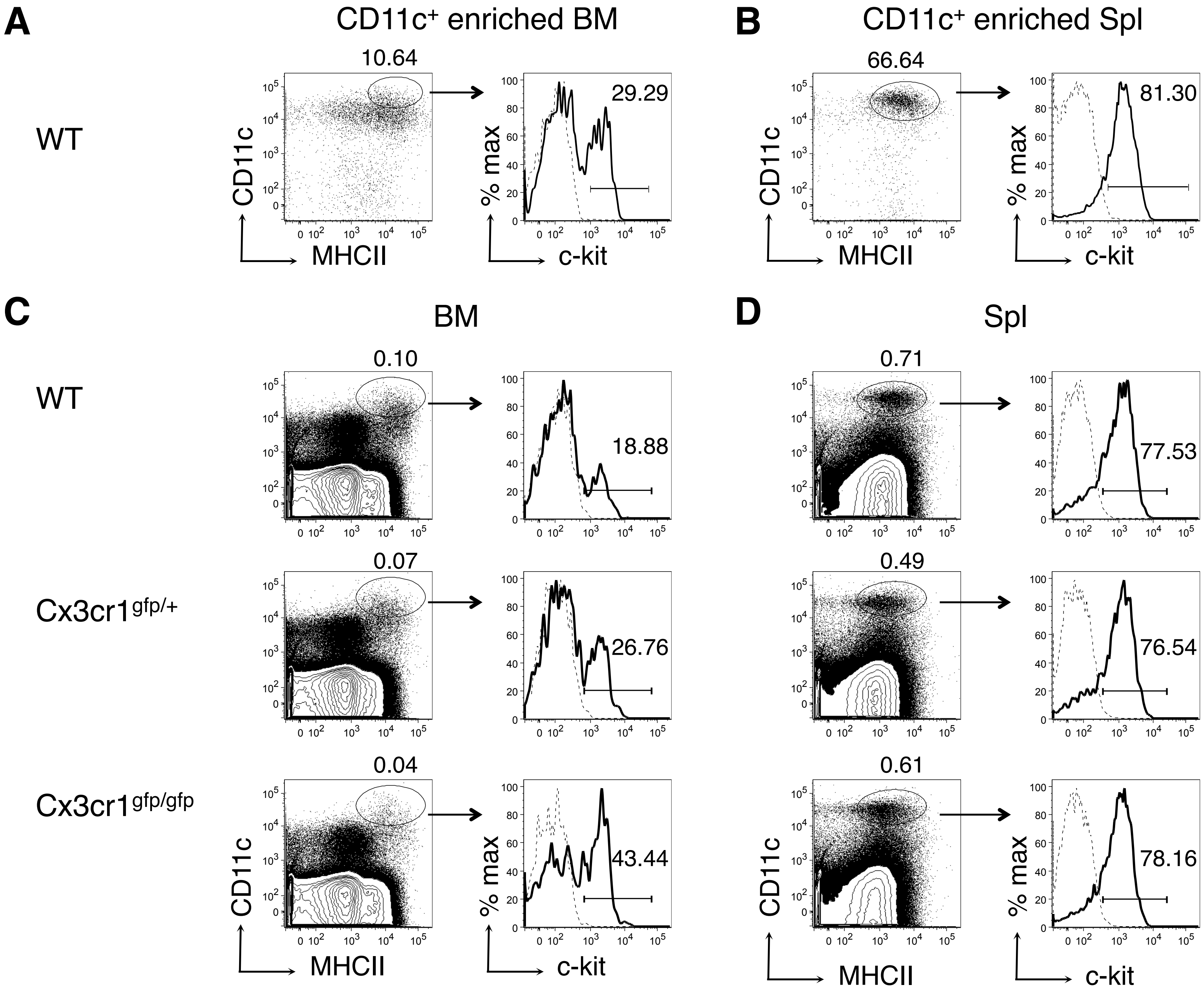
A
CD11c+ enriched BM
B
CD11c+ enriched Spl
WT
10.64
29.29
66.64
81.30
C
BM
D
Spl
WT
0.10
18.88
0.71
77.53
Cx3cr1gfp/+
0.07
26.76
0.49
76.54
Cx3cr1gfp/gfp
0.04
43.44
0.61
78.16
CD11c
MHCII
% max
c-kit

Fig. S2

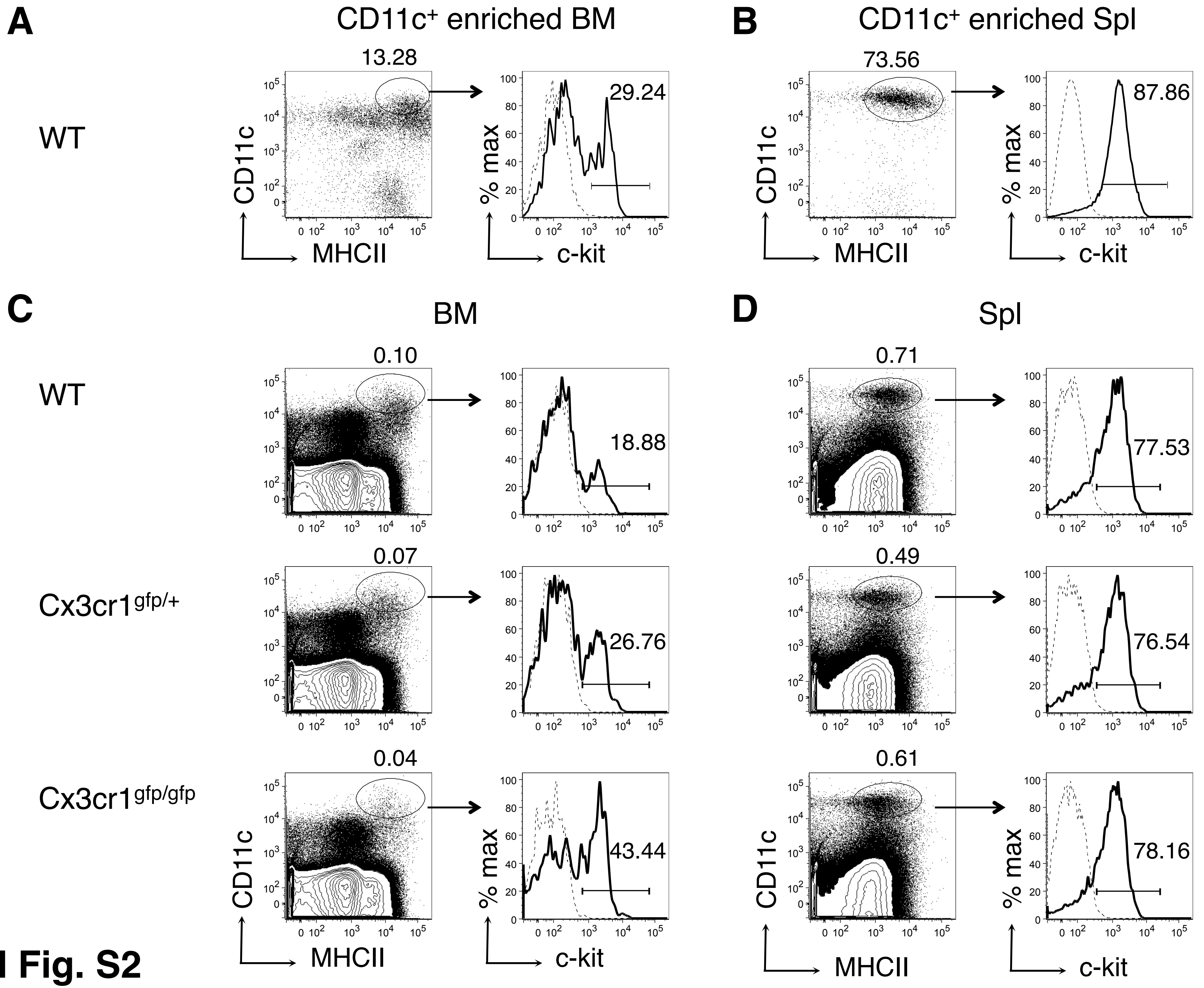

Revised Fig. S2
